# A systems biology approach to construct the gene regulatory network of systemic inflammation via microarray and databases mining

**DOI:** 10.1186/1755-8794-1-46

**Published:** 2008-09-30

**Authors:** Bor-Sen Chen, Shih-Kuang Yang, Chung-Yu Lan, Yung-Jen Chuang

**Affiliations:** 1 Lab of Control and Systems Biology, Department of Electrical Engineering, National Tsing Hua University, Hsinchu, 300, Taiwan; 2 Department of Life Science, National Tsing Hua University, Hsinchu, 300, Taiwan

## Abstract

**Background:**

Inflammation is a hallmark of many human diseases. Elucidating the mechanisms underlying systemic inflammation has long been an important topic in basic and clinical research. When primary pathogenetic events remains unclear due to its immense complexity, construction and analysis of the gene regulatory network of inflammation at times becomes the best way to understand the detrimental effects of disease. However, it is difficult to recognize and evaluate relevant biological processes from the huge quantities of experimental data. It is hence appealing to find an algorithm which can generate a gene regulatory network of systemic inflammation from high-throughput genomic studies of human diseases. Such network will be essential for us to extract valuable information from the complex and chaotic network under diseased conditions.

**Results:**

In this study, we construct a gene regulatory network of inflammation using data extracted from the Ensembl and JASPAR databases. We also integrate and apply a number of systematic algorithms like *cross correlation threshold*, *maximum likelihood estimation method *and *Akaike Information Criterion *(AIC) on time-lapsed microarray data to refine the genome-wide transcriptional regulatory network in response to bacterial endotoxins in the context of dynamic activated genes, which are regulated by transcription factors (TFs) such as NF-*κ*B. This systematic approach is used to investigate the stochastic interaction represented by the dynamic leukocyte gene expression profiles of human subject exposed to an inflammatory stimulus (bacterial endotoxin). Based on the kinetic parameters of the dynamic gene regulatory network, we identify important properties (such as susceptibility to infection) of the immune system, which may be useful for translational research. Finally, robustness of the inflammatory gene network is also inferred by analyzing the hubs and "weak ties" structures of the gene network.

**Conclusion:**

In this study, Data mining and dynamic network analyses were integrated to examine the gene regulatory network in the inflammatory response system. Compared with previous methodologies reported in the literatures, the proposed gene network perturbation method has shown a great improvement in analyzing the systemic inflammation.

## Background

Recently, the employment of microarray technology has rapidly produced vast catalogs of gene expression activities. The immense data highlights the need for a systematic tool to identify and analyze the underlying gene regulatory networks [1,2]. Several computational methods for the inference of transcriptional regulatory networks from experimental microarray data in Saccharomyces cerevisiae have been published [3,4]. The genome-wide transcriptional responses of inflammation are usually focused on the known functional interactions of the master switch proteins, such as Rel or NF-*κ*B proteins [5-7]. The identification of NF-*κ*B as a key player in the pathogenesis of inflammation suggests that NF-*κ*B-targeted therapeutics might be effective in treating diseases like rheumatoid arthritis (RA), which is a well-known disease where inflammatory response is causing the primary damage [8]. However, inflammation is usually a life-preserving response, as reflected by the increased risk of grave infections in people with genetic deficiencies in key components of the inflammatory signaling pathways [9].

Although inflammation is a hallmark of many human diseases [10,25], few studies have evaluated the genome-wide responses induced by systemic inflammation in human. DNA microarray has allowed the semi-quantitative measurement of gene expression programming in great depth and on a broad scale. However, it is a challenge to overcome the difficulties of recognizing and evaluating relevant biological processes from vast quantities of experimental data. Recently, systems biology has gained much attention due to emerging experimental and computation methods [1,2]. Systems biology is the coordinated study of biological systems by (1) investigating the components of networks and their interactions, (2) applying experimental high-throughput and whole-genome techniques, and (3) integrating computational methods with experimental efforts [11]. Therefore, it is more appealing to adapt a systems biology approach to study the mechanism of inflammation via high-throughput transcriptomic studies of human disease. Such systematic approach can provide insights into the regulation of immune cell activities, tolerance of innate immune system, and the susceptibility of infection in human. Based on a structured network-based approach and a statistical likelihood method, a network-based analysis of systemic inflammation in human has been given to evaluate genome-wide transcriptional responses in the context of known functional relationships among proteins, small molecules, and phenotypes [10,25]. The genome-wide interaction network is probed to identify functional modules that are perturbed in response to endotoxin exposure. A dynamic Bayesian network approach has also been developed to predict the gene regulatory networks from time course expression data [12].

Gene expression is transcriptionally controlled by inducible transcription factors. The transcription factor NF-*κ*B in particularly is pivotal in the regulation of inflammation. For example, unstimulated macrophage is kept under an inactivated condition, its NF-*κ*B is retained in the cytoplasm through interaction with inhibitory proteins known as I*κ*B. Cell stimulation by bacterial endotoxin will trigger a signaling pathway which results in the degradation of I*κ*B, leading to nuclear translocation of NF-*κ*B and activation of the transcription of various proinflammatory cytokines [13] (IL1A, IL1B, TNFA, IL6, IL8,...etc). Many crosstalks among the signaling pathways are recognized. It is now known that the biological functions of IL1A and TNFA overlap and complement with each others [4,14]. Thus, blocking only one mediator may not effectively reduce the overall inflammatory responses. Both IL1B and TNFA produce effects at an early stage of inflammation and the use of their inhibitory reagents at the later stage may not be able to reverse the most damaging events initiated by them. As a result, IL1B and TNFA may not represent the best targets for intervention in systemic inflammatory response. In another study [15], TNFA and IL1 were shown to have positive feedback loops to TNFR and IL1R, respectively. On the other hand, the NF-*κ*B also initiate the transcription of an inhibitory protein (A20) which can inactivate NF-*κ*B by suppressive phosphorylation in IKK (.(.([16]. The other important receptors in the immune system, TLR family members (TLR2 and TLR4), which recognize pathogens by means of conserved structural features of the microbes such as LPS for Gram-negative bacteria, would involve in activating the MyD88/IRAK signaling cascade, which bifurcates and leads to NF-kB and c-Jun/ATF2/TCF activation [17].

Because microarray data contain vast cataloged patterns of dynamic expression of the activated genes, we need systematic tools to identify the interaction architecture and the dynamics of the underlying gene networks. Indeed, the system identification problem of the underlying dynamic gene networks falls naturally into the category of reverse engineering [12]; a complex genetic network underlies a mass set of gene expression data, and the task is to infer the connectivity of gene circuit through dynamic gene regulatory model [11]. Therefore, to understand complex gene networks requires the integration of microarray data and dynamic modeling by a systematic approach. The systematic approach has to include computational dynamic modeling coupled with microarray data, data mining, dynamic view of rapid responses and network structural view arising from high-throughput analysis of the interacting species [18]. To achieve this, a dynamic Bayesian network (DBN) method has been developed to predict gene regulatory networks from time series data [12]. However, this study has not combined with other network algorithms and knowledge-based databases. It carries two fundamental problems which greatly reduce the effectiveness of the DBN approach. The first problem is the relatively low accuracy of prediction inherently, and the second is the excessive computation time.

Since the identification of a perturbed biological networks under the effect of bacterial endotoxin is an important topic in basic and clinical research, it is imperative to conduct systematic analysis based on the expression profiles of microarray data. An approach of combining genome-wide expression analysis with a clustering method has been introduced to identify functional networks using a GRAM (Genetic Regulatory Modules) algorithm to provide biological insights into gene regulatory networks [19]. Because the clustering algorithms are employed to identify sets of co-expressed and potentially co-regulated genes from gene expression data, it is more suitable to find a gene module as a set of co-expressed genes to which the same set of transcription factors will bind to their promoter regions. Therefore, it is not suitable to construct the transcriptional regulatory networks as a dynamic model. It is hence essential to provide a new way to identify the perturbed biological networks. To achieve this, systems biology and computational biology methods will need to be employed to describe the biological functions from a dynamic systems perspective [20,21].

In our present study, a systems biology approach is proposed to achieve a gradual refinement of inflammatory regulatory network. In our study, we first construct a rough gene regulatory network of inflammation by information extracted from the Ensembl database  and JASPAR  algorithms. We then build a dynamic regulatory model according to the rough gene network with consideration of time-delay between regulatory gene and target gene to describe the gene regulatory network. Based on the dynamic regulatory model and microarray data in [10,25], a maximum likelihood method is used to identify the regulatory parameters of upstream regulatory genes for each target gene. Finally, we prune away the insignificant regulatory genes by AIC model order detection method in system identification [22] to refine the gene regulatory network of inflammatory response to bacterial endotoxin. By comparing with normal gene regulatory networks, we obtain the perturbed gene network to analyze the effect of inflammatory stimulus on the immune system. The hubs and "weak ties" are also discussed for the robust inflammatory gene network. Our study is also based on databases mining to construct a rough inflammatory regulatory network.

## Results

### Construction of Rough Gene Regulatory Network of Inflammation

**Figure 1:**
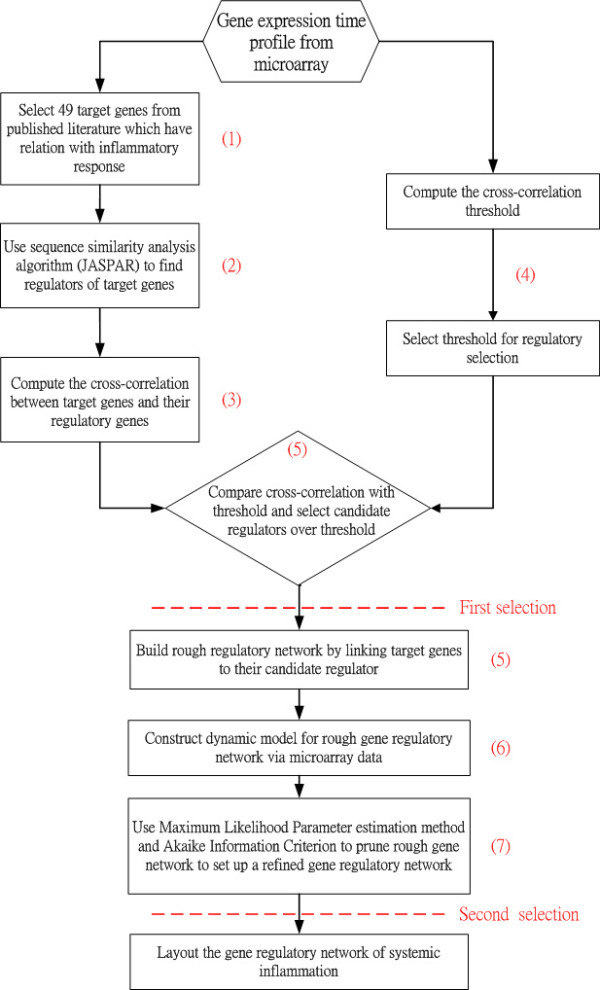
**The flow chart for constructing the gene regulatory network of inflammation**. The left-hand-side path selects target genes and their potential regulatory genes, and the right-hand-side path generates a threshold of Cross correlation between each target gene and its upstream regulator to select possible regulatory genes from the left-hand-side path to construct a rough gene regulatory network of inflammatory response. Then the rough gene regulatory network is pruned by dynamic model and parsimonious Akaike Information Criterion to achieve a refined gene regulatory network of inflammation.

The construction procedure for a gene regulatory network of inflammatory system can be divided into 7 steps in our approach (see Figure 1). The rough gene regulatory network of inflammation is set up from step 1 to step 5, and the refinement is then performed from step 5 to step 7. The step numbers are marked alongside the blocks in the flow chart.

#### Step 1

We first select 49 genes (see Table 1) that are associated with the inflammatory responses based on data mining in the published literature [10,25]. Next, we cross-reference the findings reported in other literatures [5-9], and select the candidate genes that we are interested in with bio-functions like cell-cell signaling (IL17C etc.), leukocyte migration (SCYE1 etc.) or detection of abiotic stimulus (TACR1 etc.) as candidates. (The annotations of different biological processes from *Gene Ontology database *for these 49 genes are shown in the supplemental material [see Additional file 1].) In order to distill the essence from the complicated global inflammatory gene network, we choose not to classify its function modules like Calvano et al have done in their study [10]. Instead, we only select 49 significant genes as a core in the inflammatory network, it becomes much easier to identify the permutations between normal and inflammatory conditions. It can also enable us to give biological function interpretations and to perform literature validations, especially on the NF-kB sub-network.

**Table 1:** Total 49 genes selected from published literatures

**Gene Name**	**Description**	**Gene Name**	**Description**
**ABCF1**	ATP-binding cassette, sub-family F (GCN20), member 1	**IL22**	interleukin 22
**ADORA2A**	Adenosine A2a receptor	**IL6**	interleukin 6
**ADORA3**	Adenosine A3 receptor	**IL8**	interleukin 8
**ALOX5**	Arachidonate 5-lipoxygenase	**IRAK**	Interleukin-1 receptor-associated kinase 1
**AMBP**	Alpha-1-microglobulin/bikunin precursor	**ITGB2**	integrin, beta 2
**ANXA1**	Annexin A1	**KNG**	kininogen
**AOAH**	Acyloxyacyl hydrolase (neutrophil)	**MAPK10**	mitogen-activated protein kinase 10
**BLNK**	B-cell linker	**NFATC3**	Nuclear factor of activated T-cells, cytoplasmic, calcineurin-dependent 3
**CCL18**	Chemokine (C-C motif) ligand 18 (pulmonary and activation-regulated)	**NFKB1**	Nuclear factor of kappa light polypeptide gene enhancer in B-cells 1 (p105)
**CCR7**	Chemokine (C-C motif) receptor 7	**NFKBIA**	nuclear factor of kappa light polypeptide gene enhancer in B-cells inhibitor, alpha
**CEBPD**	CCAAT/enhancer binding protein (C/EBP), delta	**NFRKB**	Nuclear factor related to kappa B binding protein
**CXCL14**	Chemokine (C-X-C motif) ligand 14	**NR3C1**	Nuclear receptor subfamily 3, group C, member 1 (glucocorticoid receptor)
**CXCL2**	chemokine (C-X-C motif) ligand 2	**PLA2G4B**	Phospholipase A2, group IVB (cytosolic)
**CYBB**	Cytochrome b-245, beta polypeptide (chronic granulomatous disease)	**PLAA**	phospholipase A2-activating protein
**FOS**	V-fos FBJ murine osteosarcoma viral oncogene homolog	**REG3A**	pancreatitis-associated protein
**GPR132**	G protein-coupled receptor 132	**SCCE**	kallikrein 7 (chymotryptic, stratum corneum)
**HDAC4**	Histone deacetylase 4	**SCYE1**	Small inducible cytokine subfamily E, member 1 (endothelial monocyte-activating)
**HDAC5**	Histone deacetylase 5	**TACR1**	Tachykinin receptor 1
**HDAC7A**	Histone deacetylase 7A	**TICAM2**	Toll-like receptor adaptor molecule 2
**HDAC9**	Histone deacetylase 9	**TLR4**	toll-like receptor 4
**HPSE**	heparanase	**TLR7**	toll-like receptor 7
**IL17**	interleukin 17C	**TNFA**	tumor necrosis factor
**IL1A**	interleukin 1a	**TNFR**	Tumor necrosis factor receptor superfamily member 1A precursor
**IL1B**	interleukin 1b	**TOLLIP**	Toll interacting protein
**IL1R**	Interleukin-1 receptor type I precursor		

Our goal is to select the candidate regulators (i.e. TFs) of 49 target genes in inflammatory response to construct the rough gene regulatory network of inflammation by linking these target genes to their regulators.

#### Step 2

We explore the Ensembl database  to retrieve the promoter sequences of 49 target genes and then conduct sequence similarity analysis to identity candidate regulators of these target genes in JASPAR , which is a high-quality transcription factor database. In this stage, we hypothesize that if some TFs are selected by the predictions of JASPAR using our criterions, the genes generating the respective TFs at the protein level could be considered as candidate regulators to the target genes.

After this step, we obtain a set of candidate regulators from the JASPAR analysis [see Additional file 2, column (A)]. However, there are still many false positive errors in our hits because the outcome has listed all possible regulators in conditions beyond inflammatory response. Some pruning methods based on microarray data of inflammatory response are necessary.

The pruning procedure is described after step 5.

#### Step 3

We screen and select potential regulators from the JASPAR hits by *Cross correlation threshold *of gene expression data [23], which is based on the assumption that there are possible correlations between target gene and their upstream regulators, with or without time delays. We compute the cross correlations between the target genes and their own regulatory genes separately, and the cross correlation values is then used to identify the candidate regulators according to the assumption that the regulatory genes and target genes have a positively (or negatively) correlated temporal relationship if the target gene's expression profile is positively (or negatively) correlated with the regulatory genes profile, with or without time lags.

#### Step 4

**Figure 2:**
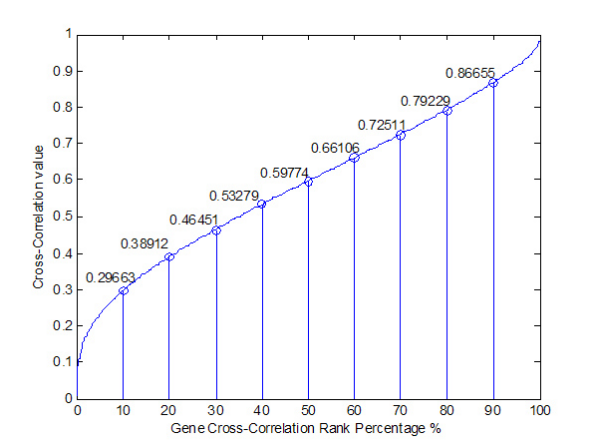
**Distribution of a threshold for selecting candidate regulators by Cross correlation method**. 2000 genes are randomly chosen from 22577 genes to compute their correlation and then these correlations are ranked. A threshold 0.3 is specified to select possible candidate regulators from those based on DNA sequence similarity in JASPAR database.

A careful choice of proper threshold for correlation to discriminate the "by chance" associations is indeed important. In order to decide on a threshold of significant correlations between transcription regulators and target genes for selection of candidate transcription regulatory genes, we randomly choose 2000 genes from 22577 genes and computed their correlations by the *Pearson Correlation *in equation (3), as ranked in Figure 2. According to the ranking in Figure 2, we select the 30% (i.e. 0.46451 in cross correlation value) as our threshold. A lower threshold may recruit some "by chance" regulator genes, while a higher threshold may result in the increase of false-negative genes. However, we lave learned from our own experience that high correlated genes may be associated due to the fact that they are co-regulated by the same regulatory genes/transcription factors. In other word, those genes that are co-regulated by a common set of genes but do not regulate each other always arise with high correlation. On the other hand, time delay in signaling pathways may mask the genes with co-regulatory association with low correlation. Because the correlation is only the first discrimination parameter used in our gradual refinement of the inflammatory regulatory network, we do not want to miss all possible candidate regulatory genes at such early stage. The aim in the first stage of our algorithm is to delete those "impossible" regulatory associations that are truly-false. Therefore, we can not adapt a high threshold of correlation for discrimination. Furthermore, by using the permutation steps for data randomization, the probability density function of parameter estimation shows the parameter estimations of regulatory genes by our threshold of correlation has a P value less than 0.001. So the genes selected by the proposed threshold (0.46451) are the significant candidates for regulatory genes.

#### Step 5

Here we make the first selection from the candidate regulators in Step 3. This implies that if the cross correlation between a candidate regulator and the target gene is more than 0.46451, it will be considered as a candidate regulator for the target gene. After selecting potential regulators by *cross correlation threshold*, these target genes and their candidate regulators are integrated to construct a preliminary gene regulatory network of inflammatory response. Results of the first selection are listed in the supplemental material [see Additional file 2, column (B)].

### Pruning the Preliminary Gene Regulatory Network via a Dynamic Model

By this point we have constructed a preliminary network via the first five selection steps using statistical inferences. However, we have yet to consider the dynamic property of this network. To include the dynamic parameters, we apply the *Akaike Information Criterion *(AIC), to help us make a more comprehensive selection. The AIC algorithm is denoted as Step 7 in Figure 1. A dynamic regulatory model is proposed to parsimoniously describe the gene regulatory genetic network of inflammation. It should be mentioned that the time delays from the regulators to their target genes, which have been detected by *Cross correlation prediction algorithm *via correlation, are considered in the dynamic regulatory model to mimic the delay phenomenon due to the transduction relay of the metabolic and signal pathways in the real transcriptional regulatory process. Details of the pruning are presented in the following paragraph and in the Material and Methods section.

In this study, based on the possible interactions in a preliminary gene regulatory network of inflammation [10,24,25] obtained from the previous sections, a dynamic regulatory model for the transcription of an interested target gene of systemic inflammation is developed. This model describes how the upstream regulatory genes control their target genes to produce the output expression of mRNA through transcriptional regulatory network. From the rough gene network through database-predicted information, we construct a dynamic regulatory model for each target gene of systemic inflammation in humans. Then, according to the microarray data of genetic expression, we identify the number of connections in the dynamic regulatory model of rough gene network in the inflammatory system. Based on the degree of interaction in the regulatory network, we prune the preliminary gene regulatory network of inflammation one target gene at a time via *Akaike Information Criteria *(AIC). The pruning procedures to obtain a refined gene regulatory network (see Figure 1) are given in the following steps.

#### Step 6

According to the rough gene network, the transcriptional regulation of a target gene in inflammatory system is dynamically modeled in the following multi-input/single-output stochastic process.

(1) $$y(t+1)=ay(t)+\sum_{i=1}^{L}b_{i}x_{i}(t-\tau_{i})+k+\varepsilon (t)$$

where *y*(*t*) represents mRNA expression level of a target gene at time *t*, and parameter *a *indicates the effect of the present state *y*(*t*) on the next state *y*(*t *+ 1); *x*_*i*_(*t *- *τ*_*i*_), i = 1,..., L, denotes the regulation functions of L upstream transcription factors in the rough gene network; and *b*_*i*_, i = 1,..., L denotes their corresponding kinetic coefficients (or regulation abilities). In addition, *τ*_*i *_denotes the expression delay from regulatory gene i to the target gene, which was detected via identifying the model by the fact that at the delay *τ*_*i *_regulatory gene i has the highest correlation with the target gene. The value of *τ*_*i *_will be iteratively detected from 0 to 2 hours (4 time points) by a minimum loss function based on AIC in the final pruning step (AIC). It can be ensured that the *τ*_*i *_value we detect has the best model fitting, although it has a large amount of computations. *k *in equation (1) represents the basal molecular level to denote the regulation of unknown factors. *ε *(*t*) denotes a stochastic noise due to model uncertainty and fluctuation of the mRNA microarray in the target gene. The binding transcriptional regulatory functions *x*_*i*_(*t*) of TFs on their motif binding sites are described by the following sigmoid functions of mRNA expression profiles of their corresponding regulatory genes, respectively [26]

(2) $$x_{i}(t)=f_{i}(y_{i}(t))=\frac{1}{1+\exp(-r(y_{i}(t)-m_{i}))}$$

i.e., the sigmoid functions in equations (2) denote the thresholds of bindings of TFs on motif binding sites for the transcriptional regulation in equation (1).

#### Step 7

By combining the maximum likelihood parameter estimation method with the most parsimonious model order detection using the Akaike Information Criterion (AIC) (see Materials and Methods), we could prune the rough gene network to generate a more refined gene network through the most parsimonious gene transcription regulatory model in equation (1) i.e., the insignificant interactions (or small *b*_*i*_) could be deleted by AIC. With the upstream regulatory genes as target genes, we can then trace back their upstream regulatory genes by a similar construction procedure. Iteratively, we could construct the whole gene regulatory network of systemic inflammation in the innate immune system. The results of selection are listed [see Additional file 2, column (C)].

### Construction of inflammatory gene network in immune system

**Figure 3:**
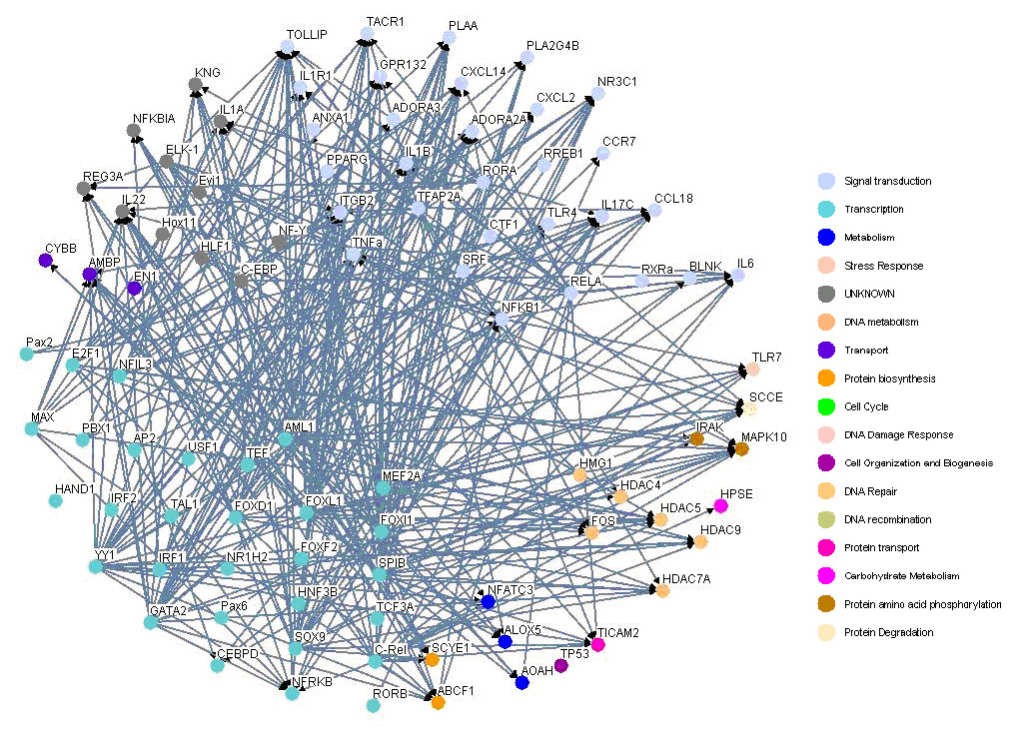
**The inflammatory transcriptional gene network in immune system with LPS**. The inflammatory gene network with LPS containing.

**Figure 4:**
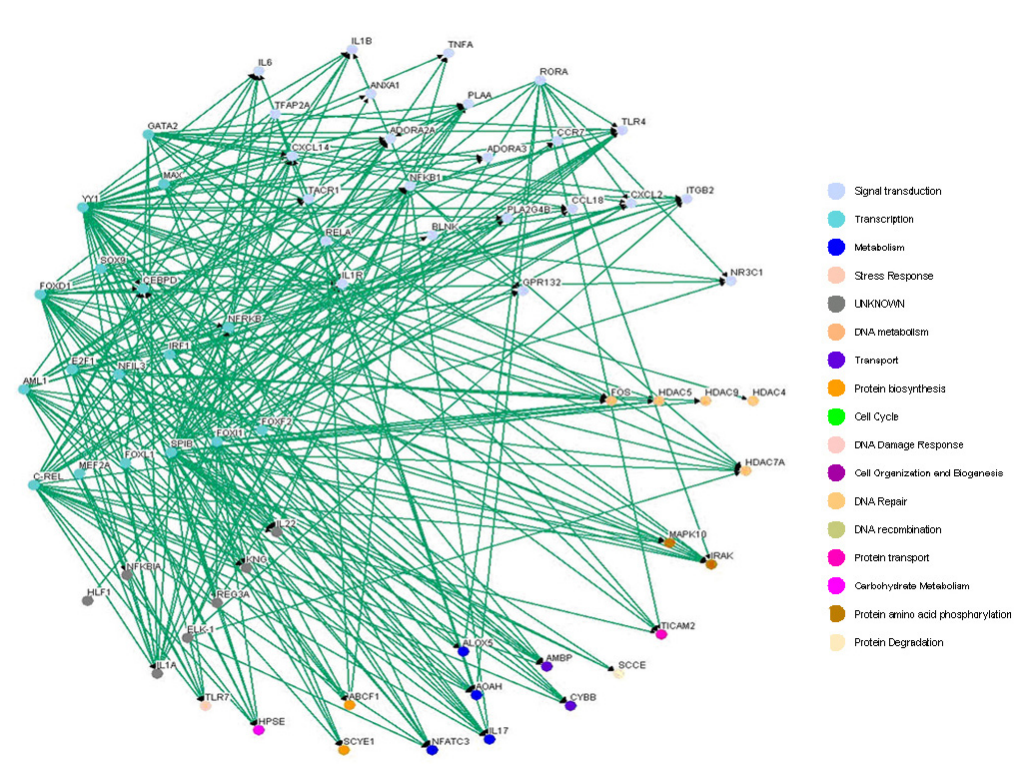
**The inflammatory transcriptional gene network in immune system without LPS**. The inflammatory gene network in normal condition.

Based on the 49 target genes (see Table 1) and their candidate regulators [see Additional file 2, Column (C)], we construct a rough gene regulatory network of the human inflammatory system. Then, according to the rough gene regulatory network, we set up the dynamic model for the rough gene regulatory network to prune it once more to set up a refined gene regulatory network by a system identification scheme and parsimonious AIC method via microarray data. At this point, we can construct two more refined gene regulatory networks for both the inflammatory/activated and the normal/resting conditions by the same construction flow chart shown in Figure 1, and draw two gene regulatory networks by the Osprey tool [27] (See Figure 3 and 4). In Figure 3, there are 94 nodes with 336 edges for the inflammatory/activated gene network and in Figure 4, there are 66 nodes with 264 edges for the normal/resting gene network.

**Figure 5:**
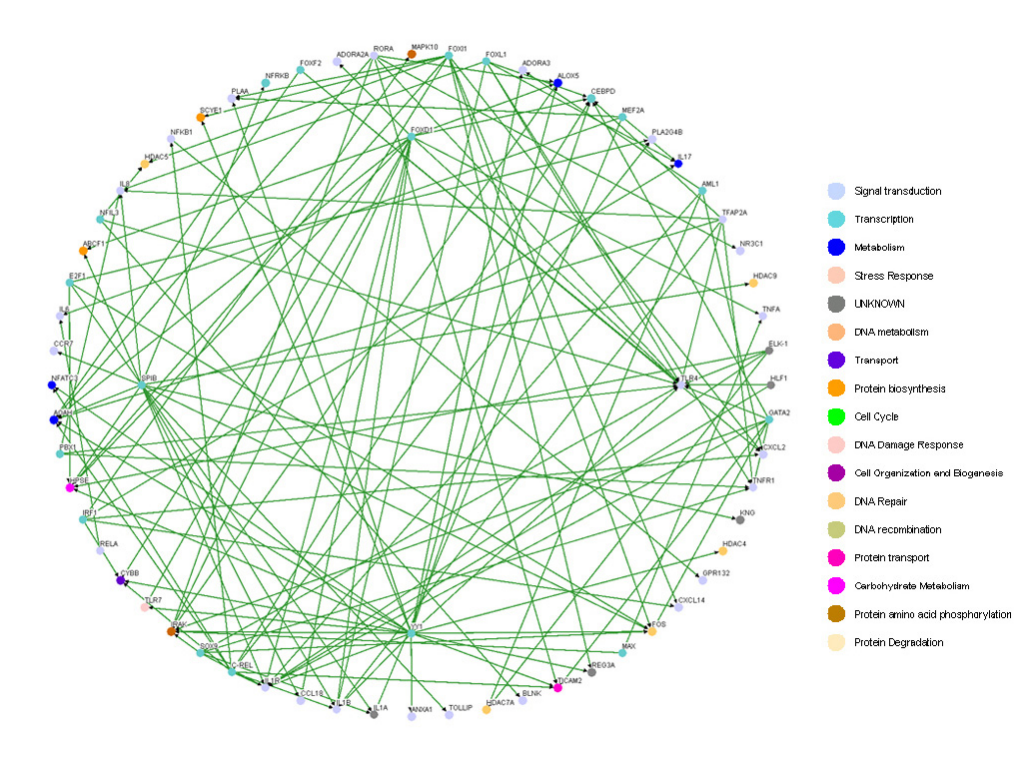
**The perturbed transcriptional gene network**. Gene network only in normal condition but not inflammatory condition.

**Figure 6:**
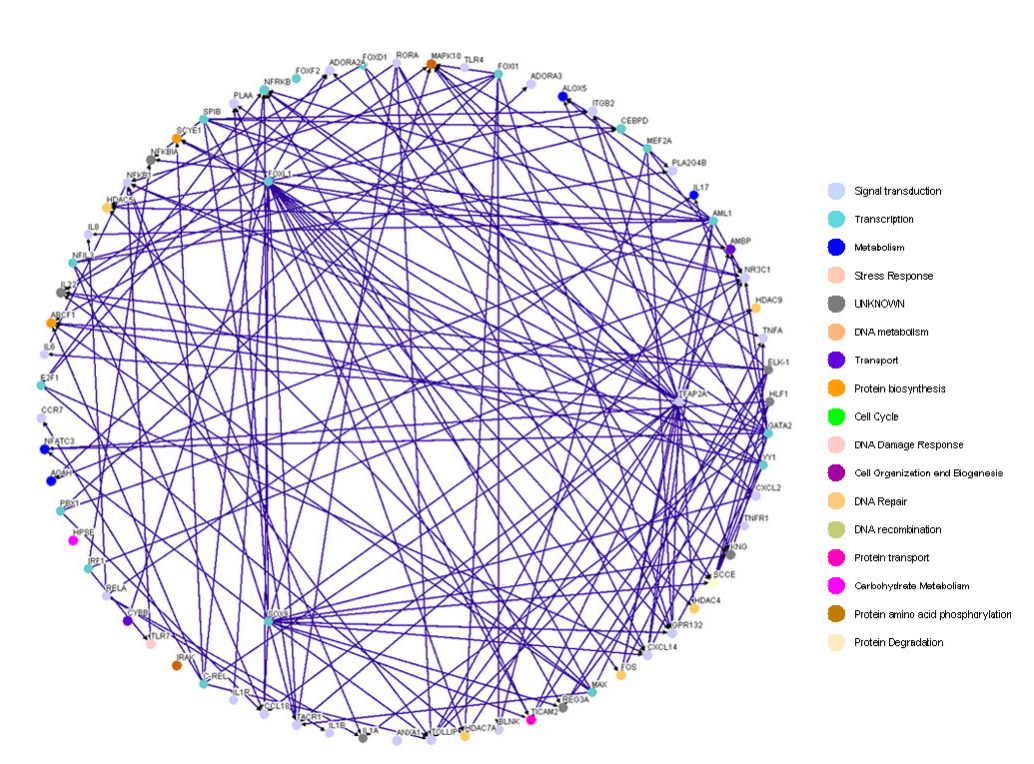
**The perturbed transcriptional gene network**. Gene network only in inflammatory condition but not in normal condition.

By comparing the inflammatory network with the normal network, we obtain the differential/perturbed gene regulatory network (see Figure 5 and 6). While some interactions can be found in both the normal and the inflammatory (LPS-treated) networks, we extracted the unique connections which only existed in one specific network but not in another. We showed the similarities and the differences in the gene regulatory network of an inflamed system between the normal and inflammatory cells [see Additional file 3]. This significant finding helps to better understand the effect of inflammatory stimulus on the innate immune system. As noted, the perturbed inflammatory gene regulatory network in the immune system between normal and LPS-stress cells is the focus of this study.

We further lay out the perturbed inflammatory gene regulatory network to locate the significance differential connection of the key components. We can observe many differences in normal and inflammatory conditions from the perturbed gene network. In Figure 5, the gene network contains 64 nodes with 131 edges found only in normal condition but not in inflammatory condition, and there are 4 hubs (FOXD1, SPIB, YY1 and TLR4) which appear to be highly connected. In Figure 6, the gene network contains 70 nodes with 159 edges for gene network found only in inflammatory condition but not in normal condition, and there are clearly 3 hubs (FOXL1, TFAP2A and SOX9) within this perturbed network. It is noteworthy that these highly connected hubs have been mentioned in several previous studies. For example, TFAP2A is inactivated [28] and SOX9 is inhibitive [29] in response to inflammation as shown in Figure 6. And FOXL1 is dramatically induced during hepatic stellate cell activation [30] and preliminary experimental data indicates that FoxL1 is involved in the regulation of the adhesion molecule ICAM-1, an important mediator of neutrophil recruitment in liver injury. The current investigation is focused on delineating the mechanism by which FOXL1 regulates inflammatory signaling in the liver.

We summarize the connection degree (i.e. the number of connections) of each node of Figure 6 in the supplemental material [see Additional file 4] and compile a list of regulators with connection degree ≧ 8 (see Table 2) to identify perturbed hub proteins that induce differences between inflammatory and normal conditions. These proteins are possible target regulators for drug discovery investigation (such as anti-inflammatory drugs [31-33]). Finally, we summarize the gene connectivity of 6 regulators (FOXL, TFAP2A, SOX9, GATA2, AML1 and NR3C1) with high degree of connectivity in Table 2, which are confirmed and in agreement with previous research findings [28-35].

**Table 2:** Gene Connectivity only in inflammatory condition but not in normal condition

Regulator	Connectivity	Reference
FOXL1	23	[17]
TFAP2A	19	[28]
SOX9	16	[29]
GATA2	12	[31,33]
AML1	11	[34]
NR3C1	8	[32,35]

It has been shown that the robust gene network can form a scale-free network, i.e. genes prefer to form links with other genes that already has highest number of links [36,37]. Scale-free gene networks could tolerate random removal of nodes but are vulnerable to loss of highly interactive hubs [36,37]. This may result in the lethal outcome in a system's behavior when highly connected hubs are targeted. In the inflammatory gene network shown in Fig. 3(A), genes such as NF-kB, TNF-α, RELA, etc. can be considered as highly connected hubs of the signaling transduction. If they are inactivated by mutation or disease, the inflammatory gene network will lead to eventual collapse of the system. In order to overcome this lethal outcome, "weak linkage" architectures are evolved by nature selection to improve the robustness of gene regulatory networks. We argue such versatile mechanisms underlie the essential regulatory process of robust gene network. As a result, some connections can easily be removed and some connections can easily be added in the gene regulatory network. Such concept is also known as "weak ties" in network theory [37]. "Weak ties" structures in biological networks enable remove of old processes and addition of new processes to the existing core processing to improve the information exchanges and signal transductions using common versatile mechanisms that operate on diverse inputs to various stimuli [36]. As a consequence, "weak ties" can improve network's robustness against external stimuli. Obviously, the connections of the perturbed gene network in Fig. 4(a) are presented only in the normal condition. The perturbed gene network in Fig. 4(b) can hence be considered as additional connections in the inflammatory gene network. In response to bacterial endotoxin, the connections in Fig. 4(a) are removed and the connections in Fig. 4(b) are added. Apparently, this agrees with the concept of the so-called "strength of weak ties" in network theory, where the most important interactions and information exchanges sometimes occur via nodes from otherwise unrelated networks. This implies that non-hubs may play a pivotal role in the gene regulation [36,37]. Similarly, the "weak ties" architecture in NF-kB gene network in inflammatory condition is shown in the removal and addition of connections of gene network in Fig. 6(a) and Fig. 6(b).

In summary, the regulators of target genes are first selected by JASPAR, then truncated by the threshold of Cross correlation and finally pruned by AIC via microarray data and a dynamic model. We combine several algorithms and tools to improve the performance of the gene network construction of the target inflammatory system. All the data sources are independently produced by various research groups and the results are verified with more independent studies published previously. It is clear that the top-down procedures can predict the target genes and their candidate regulatory transcription factors well. More biological insight into the perturbed inflammatory network is given in the Discussion section below and details of the proposed gene regulatory network construction algorithm are shown in Material and Methods.

## Discussion

**Figure 7:**
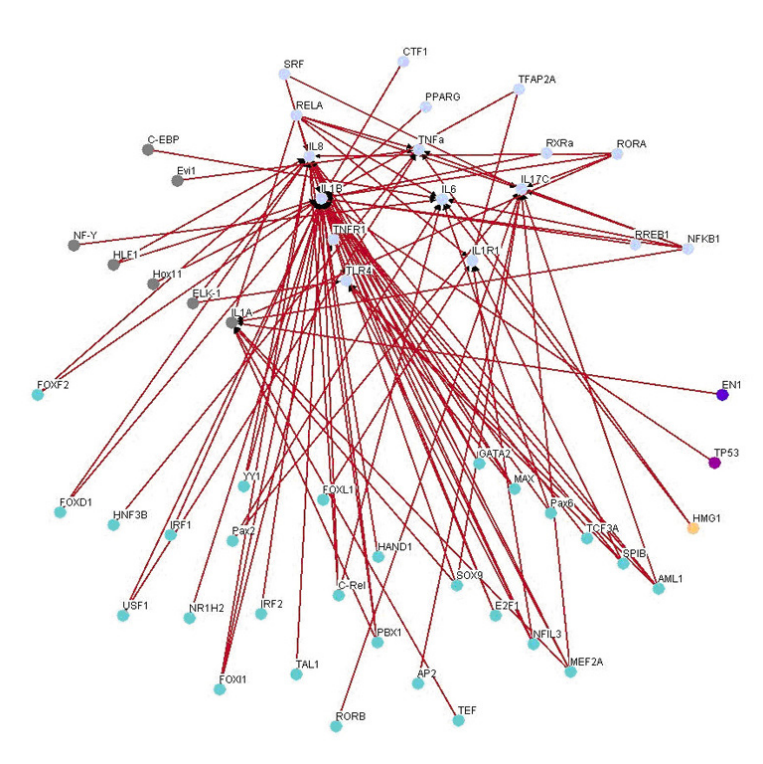
**The important proinflammatory gene network induced or activated by NF-*κ*B in immune system with LPS**. The important proinflammatory gene network in inflammatory condition.

**Figure 8:**
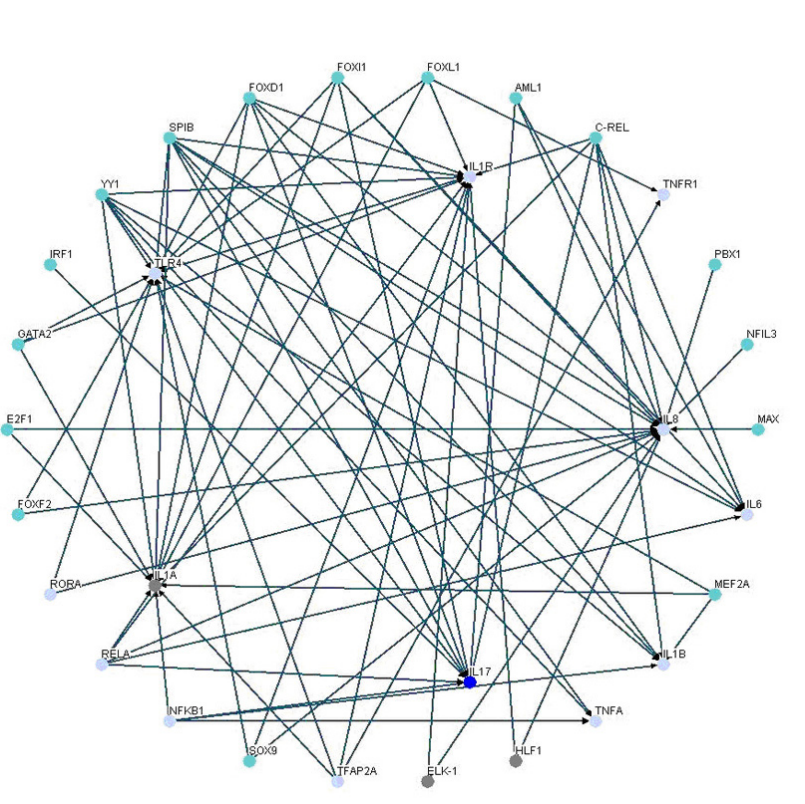
**The important proinflammatory gene network induced or activated by NF-*κ*B in immune system without LPS**. The important proinflammatory gene network in normal condition.

The NF-*κ*B pathway, which is an important modular inflammatory system, is illustrated as a trimmed down gene regulatory network depicted in Figure 7 and 8. This concise network includes important proinflammatory cytokine genes: IL1A, IL1B, IL1R, IL6, TNFA, IL17, IL8 and the receptor genes IL1R, TLR4 and TNFR, all of which have well-known roles in the NF-*κ*B signaling pathway. This concise network can help us to monitor the performance of inflammatory responses under diverse conditions. By the proposed method shown in this study, we can predict the dynamic profiles of those cytokines. As expected, our results are comparable to the findings published in previous studies [8,24] discussed in the following paragraphs. Our *in silico *findings confirm the wet-bench observation that many characterized genes in the common inflammation response are regulated by the transcription factor NF-*κ*B [6].

**Figure 9:**
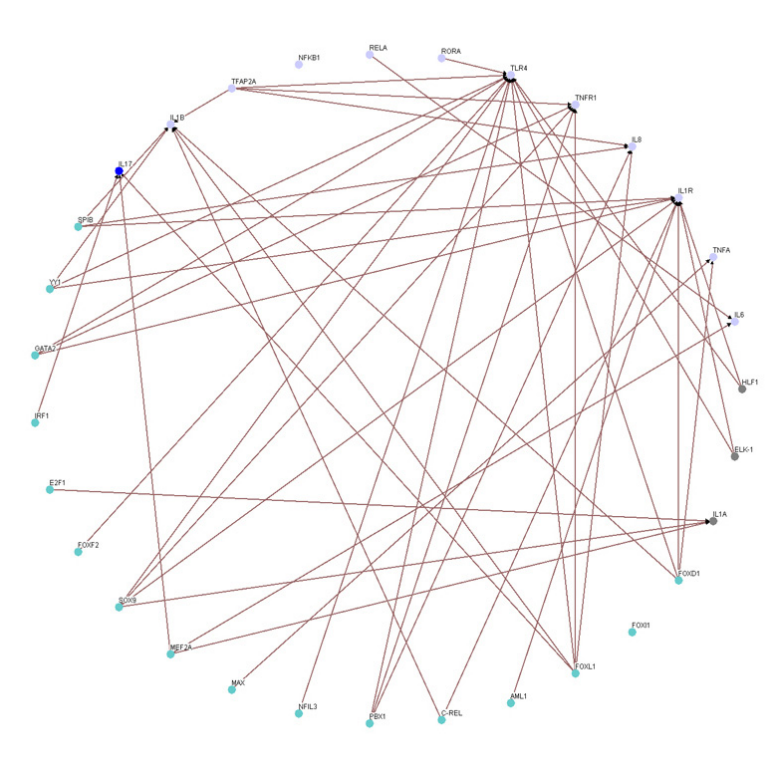
**The important proinflammatory perturbed NF-*κ*B gene network**. Gene network only in normal condition but not inflammatory condition.

**Figure 10:**
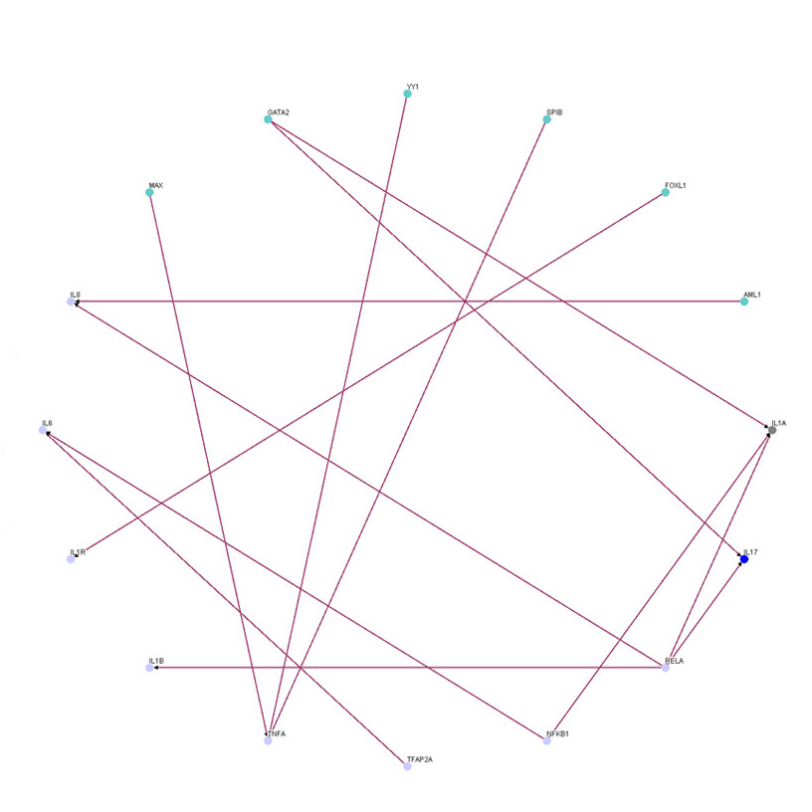
**The important proinflammatory perturbed NF-*κ*B gene network**. Gene network only in inflammatory condition but not in normal condition.

On the other hand, the perturbed gene network of these proinflammatory genes in NF-*κ*B signaling pathway is shown in Figure 9 and 10. The perturbation in Figure 9 is more complicated than the perturbation shown in Figure 10 because in normal condition these genes have to fulfill other biochemical tasks other than inflammation. Our analysis also reveals that the important genes (IL1A, IL1B, IL1R, IL6, TNFA, IL17, IL8, IL1R, TLR4 and TNFR) detected by our algorithms are vital for the inflammatory response because they are more connected during inflammation than in normal conditions. In inflamed conditions, they appear to work in accordance with each other to enhance their effects on the inflammatory responses. For example, there is strong evidences to support that NF-*κ*B1 and RELA have to regulate the proinflammatory genes collectively when they are in inflammatory responses [7].

**Figure 11:**
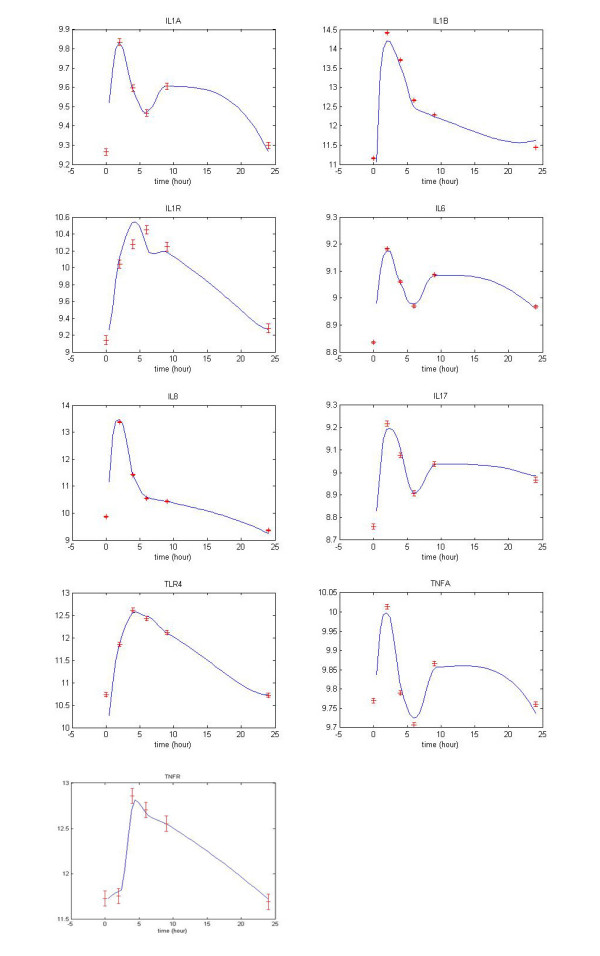
**The curve fittings of dynamic regulatory model of proinflammatory gene and its regulators**. The 'o' is the data from microarray in 24 hours and the solid line is the curve fitting by the proposed dynamic model in equation (1). The error bars for standard deviations have been marked. We denoted the curve fittings of 9 target genes and their upstream regulators respectively, and the regulatory parameters for each dynamic model are presented [see Additional file 5 and 6]

In recent studies [8,24], cytokine and chemokine networks have been shown to play a pivotal role in inflammation because they are involved either directly or indirectly in the innate and adaptive immune responses. It has been shown that Interleukin-1 alpha (IL1A) and Interleukin-1 beta (IL1B) act via their receptor (IL1R) to induce gene expressions which in term mediate a feedback protein synthesis involved in the later wave of inflammatory responses [15,24]. This is in agreement with the dynamic profiles of the proinflammatory genes and their receptors (IL1A, IL1B, IL1R, IL6, TNFA, IL17 TLR4, TNFR and IL8) which are simulated by our dynamic regulatory model (Shown in Figure 11). The accuracy of the curve fitting data has well demonstrated the prediction power of the proposed method. Without a doubt, the performance of the proposed method is very satisfactory. In Figure 11, the Interleukin-1alpha (IL1A) and Interleukin-1 beta (IL1B) have a peak expression at 2~3 hours post stimulation, and then gradually decays because of the removal of bacterial endotoxin. Interestingly, its receptor (IL-1R) has a peak expression at 6~7 hours post stimulation, while IL-1A expression has reached another peak in about 8~9 hours. This concerted changes in cytokine and receptor may be explained by the following mechanism in which IL-1A has a positive feedback loop in NF-*κ*B signaling pathway through IL-1R when the affected signaling network suffers inflammatory stress [15]. The same situations occur as well in TNF-*α *and its designated receptor TNFR. The TLR4, which is in a growing TLR family structurally characterized by a cytoplasmic Toll/interleukin-1R (TIR) domain and by extracellular leucine-rich repeats [17], has the same dynamic fluctuation as seen on IL1R or TNFR. The other genes like IL8, IL6, IL17 and their own receptors are all exhibiting similar behavior in our analyses (data not shown).

For Step 7, the identification of time delay and the estimated parameters are shown in the supplemental material [see Additional file 5 and 6]. Although we consider the effect of time lag *τ*_*i *_in our model, it is plausible that not all regulators have delay times on their transcription regulations. It seems that the regulation in inflammation may act so swiftly that parameter *τ*_*i *_can not be detected (i.e., less than one time unit of microarray data or one half hour). However, there are several time lag regulations in IL8 and its regulators, such as SOX9, MEF2A, NFIL3, ELK1, FOXF1, FOXD1, GATA2, FOXI1, REL and RELA. It is because IL8 has a more complicated regulatory mechanism through other pathways with considerable delay. The dynamic model assumes that the expression profile of a target gene results from the kinetic activity of one or more specific regulators, which bind to the downstream target gene's promoter site and initiate the transcription of that target gene to exert its effect on the inflammation network. In other words, it is possible to generate the target gene expression profile via the gene expression profiles of the upstream transcription factors using the dynamic regulatory model and its kinetic parameters in equation (1). The continuous gene expression profiles in Figure 11 [also see Additional file 5] are generated by the identified dynamic model for all target genes and their corresponding regulators, which can fit the microarray data reasonably well. Dynamic modeling of biological systems including genetic networks and cell regulatory networks has been applied in functional analyses for a long time. However, very few of the other modeling has included the time delay parameter which is comprehensively factored into our stochastic dynamic model. The findings shown in this study successfully demonstrate that we can efficiently refine the gene regulatory network of systemic inflammation in human via microarray data, and to mimic the signaling transduction delay in the transcriptional regulatory process.

Combining the cross correlation selection algorithm and the *Akaike Information Criterion*, we created a novel dynamic modeling algorithm to trim down the tangled regulatory genetic network of human inflammatory system without loss of biological meaning. The algorithm presented here can models all combinations of the target genes/regulators and produces the best predictions on gene expression by the dynamic regulatory model. Instead of attempting to model the whole complicated regulatory processes with the high risk of incorrect prediction, our dynamic model focuses only on a concise set of target genes with a more reliable outcome. Iteratively, we could eventually construct the whole gene regulatory network of systemic inflammation in response to bacterial endotoxin by our dynamic model through microarray data.

Essential problem with application of the multivariate procedures to the microarray gene expression data as expressed in recent publications is associated with reproducibility of the complex constructions resulting from such analyses. In order to confirm the reproducibility of the proposed method, we use our algorithm to rebuild the gene regulatory network via the microarray data published in reference [38]. In [38], they found there are 19 genes with significant inflammatory responses. In this situation, we reconstruct the inflammatory gene network based on these 19 genes. After comparing the reconstructed inflammatory gene regulatory network with the one in the text, we found some similarities and differences. The same highly connected hubs are GATA2, AML1 (RUNX1) and YY1. There are more than 5 connections for these hubs in both perturbed inflammatory networks. However, for the lack of some specific gene expression data in reference [38], we were unable to verify a part of highly interactive genes in the text (i.e. FOXL1, TFAP2A and SOX9). Interestingly, we also found there are some hubs only present in the reconstructed network but not in the text like GATA3 and FPR, which would be involved in host defense against bacterial infection and in the clearance of damaged cells [39]. The reason why these 19 candidate genes still discovered new hubs is because some of 19 candidate genes are not included in the previous 49 genes. For different experimental conditions, research topics and technology platforms, the data pool from different literature may be different. Therefore, the candidates of target genes we chose here differed from the text, so the computational results would not be identical [see Additional file 7].

In this study, we use multi-input/single-output regulatory model to dynamically describe our gene regulatory system (i.e. multiple regulators and one target gene) that can mimic the real gene regulation in response to inflammation. The simulation can figure out the regulatory relationship and time lag value between upstream regulator and downstream target genes using time-series microarray data. In the research of Zou et al. [12], they used the concept of time delay just in a static state analysis of gene network, without applying it to dynamic modeling to mimic the bona fide gene regulatory behavior. Furthermore, the apparent shortcoming of the static state analysis is the limitation on a single-input single-output system (i.e. one regulator and one target gene). Such single-input single-output system is rarely existed in actual gene regulation.

While significant improvement in network construction has been achieved by our method, there are still two drawbacks in this study. First, although we present a multi-input/single-output system, it still can not represent the actual biological conditions because they are multi-input/multi-output systems in most situations. This means when using AIC to trim the initial tangled gene regulatory network, we should prune down all data simultaneously rather than separately. However, such approach will increase the computational complexity in the combinatorial way and thus become computationally infeasible. The second drawback of all published algorithms for inference of transcriptional regulatory networks in inflammation, including this study, is that the candidate regulators are selected from the pool of potential regulators typically defined by computational prediction, either by sequence similarity analysis, or by other genome annotation methods. If a true regulator is not included in the pool, it will inevitably escape identification by the modeling approach. This type of error will likely become a very significant problem in a poorly characterized genome of a model organism.

## Conclusion

Our dynamic modeling represents a new approach to the study of gene regulatory network in inflammatory response. It is based on databases mining to construct an inflammatory regulatory network. It is also a systems biology approach because we process the complex regulatory network of numerous genes and regulators from various data sources at the same time. The trimmed down algorithm presented here can also be extended for global gene regulatory network analysis other than the inflammatory system in the future. From the curve fitting data generated by the proposed method, it can be seen that the performance is very satisfactory. By comparing with normal gene regulatory networks, we obtain the perturbed gene network to analyze the effect of inflammatory stimulus on the immune system. The hubs and "weak ties" are also discussed for the robust inflammatory gene network. The proposed gene regulatory network is also confirmed by published evidence in the literatures. In our future research, we will investigate the dynamic networks in a host-pathogen interaction on an animal model organism. We will also consider extending the algorithm to the identification and analysis of cross-talking transcriptional regulatory networks.

## Materials and methods

### Dataset selection

We used previous microarray data [10,25] as our mRNA expression profiles. Gene expression in whole blood leukocytes was determined at 0, 2, 4, 6, 9 and 24 hours after the intravenous administration of bacterial endotoxin to four healthy human subjects. In those experiments, four additional subjects were studied under identical conditions but without endotoxin administration. The infusion of endotoxin activates innate immune responses and presents physiological responses of brief duration. It should be noted that there is an initial proinflammatory phase and a subsequent counter-regulatory phase, with resolution of virtually all clinical perturbation within 24 hours.

### Construction of Rough Gene Networks of Systemic Inflammation

Cross correlation is developed to identify target genes that are regulated by a common set TFs. The cross correlation uses continuous gene expression with the assumption that the regulatory genes and target genes have a level of positively (negatively) temporal correlation relationship if the target gene's expression profile is positively (negatively) correlated with the regulatory gene's profile, possibly with time lags. The next procedure is to specify the threshold for the correlation between target genes and their regulators. In this study, there are 22,577 gene expression time profiles [10,25]. We choose 2000 gene expression profiles randomly and computed their correlations with different time lags or lead to evaluate a threshold for significant correlations for possible regulators of target genes, which are useful for selecting candidate regulators from those via JASPAR. Let $\vec{u}$ = (*u*_1_,..., *u*_*N*_) be the expression profile of gene *u *and $\vec{v}$ = (*v*_1_,..., *v*_*N*_) be another expression profile of gene *v*. N is the time points of expression profile. Compute the correlation between $\vec{u}$ and $\vec{v}$ with the lag or lead of *h *time points as follows:

(3) $$\begin{array}{l} \begin{array}{cc} r(h)≜\left(\sum_{i=1}^{N-h}\left(u_{i+h}-\bar{u}\right)\left(v_{i}-\bar{v}\right)\right)/\left(\sqrt{\sum_{i=1}^{N-h}{\left(u_{i+h}-\bar{u}\right)}^{2}}\cdot \sqrt{\sum_{i=1}^{N-h}v_{i}-{\bar{v}}^{2}}\right), & h=-M,...,0,...,M \end{array}\\ \begin{array}{cc} \bar{u}≜\left(\sum_{i=1}^{N-h}u_{i+h}\right)/\left(N-h\right), & \bar{v}≜\left(\sum_{i=1}^{N-h}v\right)/\left(N-h\right) \end{array} \end{array}$$

Here *M *is the maximal time lead or lag between each two genes. Because we initially do not know which are the target genes and which are the regulator genes. Since each time-interval in *h *is a half-hour, we allow 2 hours lead and lag and compute the correlation between a gene and a TF with all possible time lags or leads that are less than 2 hours for regulatory response.

Finally, we select the maximum correlation between two genes with different time delays or time leads as their correlation and rank them in Figure 2 for all regulatory genes. We can obtain the distribution of correlation based on their ranks. Then, we can decide a threshold for a possible regulatory relationship between regulators and their target genes (see Figure 2). In this study, a correlation larger than 30% (or 0.46451) is selected as a threshold for possible regulators, which is used to truncate all impossible regulators from the pool of regulators suggested by JASPR via DNA sequence similarity analysis. Then, we link the remainder regulators selected by cross correlation threshold with their target genes to construct a rough gene regulatory network. After the rough gene regulatory network of inflammation system is constructed by integrating target genes with their regulators selected by cross correlation, the rough gene regulatory network is modeled by dynamic equation in (1) for further pruning. Therefore, the kinetic parameters of regulatory dynamic model are identified by the maximum likelihood parameter estimation via microarray data. After parameter identification, the insignificant interaction coefficients of dynamic model are pruned by the most parsimonious Akaike Information Criterion (AIC) to refine the gene regulatory network in the inflammatory condition. The possible regulators selected by JASPAR algorithm are pruned two times in our method, once by correlation threshold via Cross correlation and again by AIC via dynamic model and microarray data. The details are described below.

### Constructing a dynamic model for gene regulatory network via microarray data

After constructing the stochastic dynamic equation in equation (1) to model the regulation of a target gene, we use the method of maximum likelihood to estimate the kinetic parameters of dynamic model. Equation (1) can be written in the following form.

(4) $$y[t+1]=\left[\begin{array}{cc} y[t] & \begin{array}{cc} x_{1}[t-\tau_{i}] & \begin{array}{cc} \cdot \cdot \cdot & x_{L}[t-\tau_{i}]\text{ 1} \end{array} \end{array} \end{array}\right]\cdot \left[\begin{array}{c} a\\ b_{1}\\ \vdots \\ b_{L}\\ k \end{array}\right]+\varepsilon [t]$$

where *ϕ*[*t*] denotes the regression vector which can be obtained from microarray data, and *θ *∈ *R*^*p *^denotes the parameter vector of dimension *p *in regression equation (4).

After applying the cubic spline method to interpolate the microarray data, we can obtain as many data points as we want. Then it is easy to obtain values of {*y *[*t *+ *l*] *x*_*i *_[*t *+ *l*]} for *l *∈ {1, 2,⋯, *m *and *i *∈ {1 2 ⋯ *L*, where *m *is the number of expression time points of a target gene, and L is the number of TFs binding to the target gene in the rough gene network. By further computation of equation (4) at different time points we can obtain the following vector form equation by data point interpolation.

(5) $$\left[\begin{array}{c} y[t+2]\\ y[t+3]\\ \vdots \\ y[t+m-1]\\ y[t+m] \end{array}\right]=\left[\begin{array}{c} \phi [t+1]\\ \phi [t+2]\\ \vdots \\ \phi [t+m-2]\\ \phi [t+m-1] \end{array}\right]\cdot \theta +\left[\begin{array}{c} \varepsilon [t+1]\\ \varepsilon [t+2]\\ \vdots \\ \varepsilon [t+m-2]\\ \varepsilon [t+m-1] \end{array}\right]$$

For simplicity, it can be represented as follows.

(6) *Y *= Φ·*θ *+ *e*

In equation (6), the random noise *ε*[*t*_*k*_] is regarded as a random variables of white Gaussian noise with zero mean and unknown variance *σ*^2^, i.e., *E*{*e*} = 0, and Σ_*e *_= *E*{*ee*^*T*^} = *σ*^2^*I*, where *I *is an identity matrix. In this study, a maximum likelihood parameter estimation method is used to estimate *θ *and *σ*^2 ^by the regression data obtained from the microarray data of regulatory genes and the target gene [34]. Under the assumption of the Gaussian noise vector *e *with *m *- 1 elements, its probability density function is given as follows.

(7) $$p(e)=\frac{1}{{\left({\left(2\pi \right)}^{m-1}\det\Sigma_{e}\right)}^{1/2}}\exp(-\frac{1}{2}e^{T}\Sigma_{e}^{-1}e)$$

From equation (7), we can obtain the likelihood function

(8) $$L(\theta ,\sigma^{2})=P(\theta ,\sigma^{2})=\frac{1}{{\left(2\pi \sigma^{2}\right)}^{(m-1)/2}}\exp\{-\frac{{\left(Χ-\Phi \cdot \theta \right)}^{T}(Χ-\Phi \cdot \theta )}{2\sigma^{2}}\}$$

Equation (8) can be considered as a function of parameters *θ *and *σ*^2^. In order to simplify the computation, it is practical to take the logarithm of equation (8), which yields the following log-likelihood function:

(9) $$\log L(\theta ,\sigma^{2})=-\frac{m-1}{2}\log(2\pi \sigma^{2})-\frac{1}{2\sigma^{2}}\sum_{k=1}^{m-1}{\left[y[t+k+1]-\phi [t+k]\cdot \theta \right]}^{2}$$

where *y *[*t *+ *k*] and *ϕ*[*t *+ *k*] are the *k-th *elements of *Y *and Φ in (6), respectively.

By the maximum likelihood parameter estimation method, we expect the log-likelihood function to have the maximum at *θ *= $\hat{\theta }$ and *θ*^2 ^= ${\hat{\sigma }}^{2}$. The necessary conditions for the maximum likelihood estimates $\hat{\theta }$ and ${\hat{\sigma }}^{2}$ are as follows [22],

(10) $$\begin{array}{l} \frac{\partial \log L(\theta ,\sigma^{2})}{\partial \theta }=0\\ \frac{\partial \log L(\theta ,\sigma^{2})}{\partial \sigma^{2}}=0 \end{array}$$

The estimated parameters $\hat{\theta }$ and ${\hat{\sigma }}^{2}$ are shown below,

(11) $$\hat{\theta }={\left(\Phi^{T}\Phi \right)}^{-1}\Phi^{T}Y$$

(12) $${\hat{\sigma }}^{2}=\frac{1}{m-1}\sum_{k=1}^{m-1}\left[y[t_{k+1}]-\phi [t_{k}]\cdot \hat{\theta }\right]=\frac{1}{m-1}{\left(Y-\Phi \cdot \hat{\theta }\right)}^{T}(Y-\Phi \cdot \hat{\theta })$$

where *Y *and Φ can be obtained from the microarray data of regulatory genes and the target gene. After obtaining the estimated parameter $\hat{\theta }$, the dynamic equation of the target gene in the estimated transcriptional regulatory network can be expressed as follows

(13) $$y[t+1]=\hat{a}\cdot y[t]+\sum_{i=1}^{L}{\hat{b}}_{i}\cdot x_{i}[t-{\hat{\tau }}_{i}]+\hat{k}+\varepsilon [t]$$

where $\hat{a}$, ${\hat{b}}_{i}$ and $\hat{k}$ are obtained from (11) and the variance of is obtained from (12).

Iteratively, one target gene at a time, we can construct the overall dynamic equations of transcriptional regulatory network of inflammation, which are interconnected through the regulations $\sum_{i=1}^{L}{\hat{b}}_{i}\cdot x_{i}[t]$ of TFs.

Since some interaction coefficients ${\hat{b}}_{i}$ of the gene regulatory network in (13) are insignificant, they should be pruned off by the parsimonious AIC criterion. This is discussed in the next section.

### Pruning the Gene Regulatory Network

First, in this study, we use the JASPAR database to identify plausible binding motifs of their TFs roughly and select candidate regulators from the pool of DNA sequence similarity analysis. A rough gene regulatory network of inflammation is constructed by linking target genes and their regulators with a cross correlation threshold larger than 30% (see Figure 2). Then we use the maximum likelihood estimation method to estimate the parameters of the dynamic model for a preliminary gene regulatory network of the inflammatory system.

Although the maximum likelihood estimation method can help us quantify the regulatory abilities of all the possible interactive candidates of regulators on target genes, we still do not know exactly how significantly the regulatory ability can be regarded as a true regulator. In order to determine whether a regulator is significant or not, a statistical approach based on model validation is proposed for evaluating the significance of our model parameters to prune the preliminary gene network. In this study, a statistical approach called the Akaike Information Criterion (AIC) is employed to validate the model order (or the number of model parameters) to determine the significance of our dynamic model parameters [22].

The Akaike Information Criterion (AIC), which attempts to include both the estimated residual variance and the model complexity in one statistic, decreases as the residual variance ${\hat{\sigma }}^{2}$ decreases and increases as the number *p *of parameters increases. As the expected residual variance decreases with increasing *p *for nonadequate model complexities, there should be a minimum around the correct number *p *of network parameters. For a transcriptional regulatory model with *p *regulatory parameters to fit with data from N samples, the Akaike Information Criterion (AIC) can be written as follows [22],

(14) $$AIC(p)=\log(\frac{1}{N}{\left(Y-\hat{Y}\right)}^{T}(Y-\hat{Y}))+\frac{2p}{N}$$

where $\hat{Y}$ denotes the estimated expression profile of the target gene, i.e. $\hat{Y}$ = *ϕ*·$\hat{\theta }$.

This is a tradeoff between residual variance and model order. The minimization of equation (14) will achieve the true model order (i.e. the number of regulators of the target gene) of the gene regulatory system [22].

After the statistical selection of *p *parameters by minimizing the Akaike Information Criterion (AIC), we can easily determine whether the regulatory TFs candidate is a significant or just a false positive and then construct a refined gene regulatory network model for inflammation. Finally, evidence from previous studies is an important validation to support our refined gene regulatory network.

## Competing interests

The authors declare that they have no competing interests.

## Authors' contributions

BSC gave the topic and suggestions and was responsible for the entire study. SKY carried out the design and computation. CYL and YJC amended and improved the design and the presentation of this study. All authors read and approved the final manuscript.

## Pre-publication history

The pre-publication history for this paper can be accessed here:



## Supplementary Material

Additional file 1 **Supplementary Table 1.** Short characteristics of 49 target genes Click here for file

Additional file 2 **Supplementary Table 2.** The inflammatory genes and their regulators Click here for file

Additional file 3 **Supplementary Table 3.** The gene regulatory network in immune system of un-activated and inflammatory cells Click here for file

Additional file 4 **Supplementary Table 4. **Gene Connectivities only in inflammatory condition but not in normal condition Click here for file

Additional file 5 **Supplementary Material S1–S9.** Identification of time delay in Step 7 Click here for file

Additional file 6 **Supplementary Table 5.** The parameters of the inflammatory gene regulator models for Additional file 1 Click here for file

Additional file 7 **Supplementary Material S10.** Reconstruction via independent data Click here for file
