# Supplementary material for: A systems biology approach to construct the gene regulatory network of systemic inflammation via microarray and databases mining
Source: BMC Med Genomics. 2008 Sep 30;1:46. doi: 10.1186/1755-8794-1-46 (PMC2567339; doi:10.1186/1755-8794-1-46)
Supplement: Additional file 1 — Supplementary Table 1. Short characteristics of 49 target genes [file 1755-8794-1-46-S1.doc]

**Supplementary Table 1:**

**Short characteristics of 49 target genes**

| **Gene Name** | **Description** | **Gene Ontology  Biological Process** |
| --- | --- | --- |
| **IL17C** | interleukin 17C | GO:0007166 : [cell surface receptor linked signal transduction](http://amigo.geneontology.org/cgi-bin/amigo/term-details.cgi?term=GO:0007166&session_id=9907amigo1216796236&)  GO:0007267 : [cell-cell signaling](http://amigo.geneontology.org/cgi-bin/amigo/term-details.cgi?term=GO:0007267&session_id=9907amigo1216796236&)  GO:0006954 : [inflammatory response](http://amigo.geneontology.org/cgi-bin/amigo/term-details.cgi?term=GO:0006954&session_id=9907amigo1216796236&) |
| **IL1A** | interleukin 1a | GO:0006954 : [inflammatory response](http://amigo.geneontology.org/cgi-bin/amigo/term-details.cgi?term=GO:0006954&session_id=4384amigo1216796108&) |
| **TNFA** | tumor necrosis factor | GO:0006959 : [humoral immune response](http://amigo.geneontology.org/cgi-bin/amigo/term-details.cgi?term=GO:0006959&session_id=1760amigo1216795687&)  GO:0006954 : [inflammatory response](http://amigo.geneontology.org/cgi-bin/amigo/term-details.cgi?term=GO:0006954&session_id=1760amigo1216795687&)  GO:0043123 : [positive regulation of I-kappaB kinase/NF-kappaB cascade](http://amigo.geneontology.org/cgi-bin/amigo/term-details.cgi?term=GO:0043123&session_id=1760amigo1216795687&)  GO:0051092 : [positive regulation of NF-kappaB transcription factor activity](http://amigo.geneontology.org/cgi-bin/amigo/term-details.cgi?term=GO:0051092&session_id=1760amigo1216795687&)  GO:0051023 : [regulation of immunoglobulin secretion](http://amigo.geneontology.org/cgi-bin/amigo/term-details.cgi?term=GO:0051023&session_id=1760amigo1216795687&) |
| **IL6** | interleukin 6 | GO:0007267 : [cell-cell signaling](http://amigo.geneontology.org/cgi-bin/amigo/term-details.cgi?term=GO:0007267&session_id=2346amigo1216796307&)  GO:0006959 : [humoral immune response](http://amigo.geneontology.org/cgi-bin/amigo/term-details.cgi?term=GO:0006959&session_id=2346amigo1216796307&)  GO:0006954 : [inflammatory response](http://amigo.geneontology.org/cgi-bin/amigo/term-details.cgi?term=GO:0006954&session_id=2346amigo1216796307&)  GO:0045727 : [positive regulation of translation](http://amigo.geneontology.org/cgi-bin/amigo/term-details.cgi?term=GO:0045727&session_id=2346amigo1216796307&) |
| **IL1B** | interleukin 1b | GO:0007267 : [cell-cell signaling](http://amigo.geneontology.org/cgi-bin/amigo/term-details.cgi?term=GO:0007267&session_id=3267amigo1216796402&)  GO:0006954 : [inflammatory response](http://amigo.geneontology.org/cgi-bin/amigo/term-details.cgi?term=GO:0006954&session_id=3267amigo1216796402&)  GO:0007165 : [signal transduction](http://amigo.geneontology.org/cgi-bin/amigo/term-details.cgi?term=GO:0007165&session_id=3267amigo1216796402&) |
| **TLR4** | toll-like receptor 4 | GO:0007249 : [I-kappaB kinase/NF-kappaB cascade](http://amigo.geneontology.org/cgi-bin/amigo/term-details.cgi?term=GO:0007249&session_id=8609amigo1216796553&)  GO:0042116 : [macrophage activation](http://amigo.geneontology.org/cgi-bin/amigo/term-details.cgi?term=GO:0042116&session_id=8609amigo1216796553&)  GO:0007165 : [signal transduction](http://amigo.geneontology.org/cgi-bin/amigo/term-details.cgi?term=GO:0007165&session_id=8609amigo1216796553&)  GO:0042088 : [T-helper 1 type immune response](http://amigo.geneontology.org/cgi-bin/amigo/term-details.cgi?term=GO:0042088&session_id=8609amigo1216796553&) |
| **NFATC3** | Nuclear factor of activated T-cells, cytoplasmic, calcineurin-dependent 3 | GO:0006954 : [inflammatory response](http://amigo.geneontology.org/cgi-bin/amigo/term-details.cgi?term=GO:0006954&session_id=1682amigo1216796661&) |
| **SCYE1** | Small inducible cytokine subfamily E, member 1 (endothelial monocyte-activating) | GO:0007267 : [cell-cell signaling](http://amigo.geneontology.org/cgi-bin/amigo/term-details.cgi?term=GO:0007267&session_id=2238amigo1216796699&)  GO:0006954 : [inflammatory response](http://amigo.geneontology.org/cgi-bin/amigo/term-details.cgi?term=GO:0006954&session_id=2238amigo1216796699&)  GO:0050900 : [leukocyte migration](http://amigo.geneontology.org/cgi-bin/amigo/term-details.cgi?term=GO:0050900&session_id=2238amigo1216796699&)  GO:0007165 : [signal transduction](http://amigo.geneontology.org/cgi-bin/amigo/term-details.cgi?term=GO:0007165&session_id=2238amigo1216796699&) |
| **TICAM2** | Toll-like receptor adaptor molecule 2 | GO:0043123 : [positive regulation of I-kappaB kinase/NF-kappaB cascade](http://amigo.geneontology.org/cgi-bin/amigo/term-details.cgi?term=GO:0043123&session_id=1875amigo1216796773&) |
| **HDAC4** | Histone deacetylase 4 | GO:0030183 : [B cell differentiation](http://amigo.geneontology.org/cgi-bin/amigo/term-details.cgi?term=GO:0030183&session_id=2012amigo1216796947&)  GO:0006954 : [inflammatory response](http://amigo.geneontology.org/cgi-bin/amigo/term-details.cgi?term=GO:0006954&session_id=2012amigo1216796947&) |
| **HDAC5** | Histone deacetylase 5 | GO:0030183 : [B cell differentiation](http://amigo.geneontology.org/cgi-bin/amigo/term-details.cgi?term=GO:0030183&session_id=7633amigo1216797002&)  GO:0006954 : [inflammatory response](http://amigo.geneontology.org/cgi-bin/amigo/term-details.cgi?term=GO:0006954&session_id=7633amigo1216797002&)  GO:0006355 : [regulation of transcription, DNA-dependent](http://amigo.geneontology.org/cgi-bin/amigo/term-details.cgi?term=GO:0006355&session_id=7633amigo1216797002&) |
| **HDAC7A** | Histone deacetylase 7A | GO:0030183 : [B cell differentiation](http://amigo.geneontology.org/cgi-bin/amigo/term-details.cgi?term=GO:0030183&session_id=2194amigo1216797052&)  GO:0006954 : [inflammatory response](http://amigo.geneontology.org/cgi-bin/amigo/term-details.cgi?term=GO:0006954&session_id=2194amigo1216797052&) |
| **HDAC9** | Histone deacetylase 9 | GO:0030183 : [B cell differentiation](http://amigo.geneontology.org/cgi-bin/amigo/term-details.cgi?term=GO:0030183&session_id=9151amigo1216797106&)  GO:0006954 : [inflammatory response](http://amigo.geneontology.org/cgi-bin/amigo/term-details.cgi?term=GO:0006954&session_id=9151amigo1216797106&) |
| **ITGB2** | integrin, beta 2 | GO:0007267 : [cell-cell signaling](http://amigo.geneontology.org/cgi-bin/amigo/term-details.cgi?term=GO:0007267&session_id=5721amigo1216797142&)  GO:0006954 : [inflammatory response](http://amigo.geneontology.org/cgi-bin/amigo/term-details.cgi?term=GO:0006954&session_id=5721amigo1216797142&)  GO:0007159 : [leukocyte adhesion](http://amigo.geneontology.org/cgi-bin/amigo/term-details.cgi?term=GO:0007159&session_id=5721amigo1216797142&)  GO:0008360 : [regulation of cell shape](http://amigo.geneontology.org/cgi-bin/amigo/term-details.cgi?term=GO:0008360&session_id=5721amigo1216797142&)  GO:0030593 : [neutrophil chemotaxis](http://amigo.geneontology.org/cgi-bin/amigo/term-details.cgi?term=GO:0030593&session_id=5721amigo1216797142&) |
| **CXCL2** | chemokine (C-X-C motif) ligand 2 | GO:0006954 : [inflammatory response](http://amigo.geneontology.org/cgi-bin/amigo/term-details.cgi?term=GO:0006954&session_id=3134amigo1216797240&) |
| **ALOX5** | Arachidonate 5-lipoxygenase | GO:0006954 : [inflammatory response](http://amigo.geneontology.org/cgi-bin/amigo/term-details.cgi?term=GO:0006954&session_id=2134amigo1216797283&)  GO:0019370 : [leukotriene biosynthetic process](http://amigo.geneontology.org/cgi-bin/amigo/term-details.cgi?term=GO:0019370&session_id=2134amigo1216797283&)  GO:0006691 : [leukotriene metabolic process](http://amigo.geneontology.org/cgi-bin/amigo/term-details.cgi?term=GO:0006691&session_id=2134amigo1216797283&) |
| **NFKBIA** | nuclear factor of kappa light polypeptide gene enhancer in B-cells inhibitor, alpha | GO:0007253 : [cytoplasmic sequestering of NF-kappaB](http://amigo.geneontology.org/cgi-bin/amigo/term-details.cgi?term=GO:0007253&session_id=5354amigo1216797397&)  GO:0042345 : [regulation of NF-kappaB import into nucleus](http://amigo.geneontology.org/cgi-bin/amigo/term-details.cgi?term=GO:0042345&session_id=5354amigo1216797397&) |
| **NR3C1** | Nuclear receptor subfamily 3, group C, member 1 (glucocorticoid receptor) | GO:0007165 : [signal transduction](http://amigo.geneontology.org/cgi-bin/amigo/term-details.cgi?term=GO:0007165&session_id=9417amigo1216797450&) |
| **CEBPD** | CCAAT/enhancer binding protein (C/EBP), delta | GO:0006366 : [transcription from RNA polymerase II promoter](http://amigo.geneontology.org/cgi-bin/amigo/term-details.cgi?term=GO:0006366&session_id=8493amigo1216799936&) |
| **ANXA1** | Annexin A1 | GO:0007166 : [cell surface receptor linked signal transduction](http://amigo.geneontology.org/cgi-bin/amigo/term-details.cgi?term=GO:0007166&session_id=9234amigo1216797623&)  GO:0006954 : [inflammatory response](http://amigo.geneontology.org/cgi-bin/amigo/term-details.cgi?term=GO:0006954&session_id=9234amigo1216797623&) |
| **CYBB** | Cytochrome b-245, beta polypeptide (chronic granulomatous disease) | GO:0006954 : [inflammatory response](http://amigo.geneontology.org/cgi-bin/amigo/term-details.cgi?term=GO:0006954&session_id=9738amigo1216797700&)  GO:0045087 : [innate immune response](http://amigo.geneontology.org/cgi-bin/amigo/term-details.cgi?term=GO:0045087&session_id=9738amigo1216797700&) |
| **AOAH** | Acyloxyacyl hydrolase (neutrophil) |  |
| **REG3A** | pancreatitis-associated protein | GO:0008283 : [cell proliferation](http://amigo.geneontology.org/cgi-bin/amigo/term-details.cgi?term=GO:0008283&session_id=9395amigo1216797780&) |
| **FOS** | V-fos FBJ murine osteosarcoma viral oncogene homolog | GO:0006954 : [inflammatory response](http://amigo.geneontology.org/cgi-bin/amigo/term-details.cgi?term=GO:0006954&session_id=3545amigo1216797819&)  GO:0006357 : [regulation of transcription from RNA polymerase II promoter](http://amigo.geneontology.org/cgi-bin/amigo/term-details.cgi?term=GO:0006357&session_id=3545amigo1216797819&) |
| **IRAK** | Interleukin-1 receptor-associated kinase 1 | GO:0007250 : [activation of NF-kappaB-inducing kinase activity](http://amigo.geneontology.org/cgi-bin/amigo/term-details.cgi?term=GO:0007250&session_id=8650amigo1216797919&)  GO:0045941 : [positive regulation of transcription](http://amigo.geneontology.org/cgi-bin/amigo/term-details.cgi?term=GO:0045941&session_id=8650amigo1216797919&)  GO:0007165 : [signal transduction](http://amigo.geneontology.org/cgi-bin/amigo/term-details.cgi?term=GO:0007165&session_id=8650amigo1216797919&) |
| **PLAA** | phospholipase A2-activating protein | GO:0007165 : [signal transduction](http://amigo.geneontology.org/cgi-bin/amigo/term-details.cgi?term=GO:0007165&session_id=428amigo1216798013&) |
| **CCR7** | Chemokine (C-C motif) receptor 7 | GO:0006954 : [inflammatory response](http://amigo.geneontology.org/cgi-bin/amigo/term-details.cgi?term=GO:0006954&session_id=7333amigo1216798054&) |
| **CXCL14** | Chemokine (C-X-C motif) ligand 14 | GO:0007267 : [cell-cell signaling](http://amigo.geneontology.org/cgi-bin/amigo/term-details.cgi?term=GO:0007267&session_id=1780amigo1216798104&)  GO:0007165 : [signal transduction](http://amigo.geneontology.org/cgi-bin/amigo/term-details.cgi?term=GO:0007165&session_id=1780amigo1216798104&) |
| **PLA2G4B** | Phospholipase A2, group IVB (cytosolic) | GO:0006954 : [inflammatory response](http://amigo.geneontology.org/cgi-bin/amigo/term-details.cgi?term=GO:0006954&session_id=4576amigo1216798143&) |
| **NFRKB** | Nuclear factor related to kappa B binding protein | GO:0006954 : [inflammatory response](http://amigo.geneontology.org/cgi-bin/amigo/term-details.cgi?term=GO:0006954&session_id=6994amigo1216798185&)  GO:0006366 : [transcription from RNA polymerase II promoter](http://amigo.geneontology.org/cgi-bin/amigo/term-details.cgi?term=GO:0006366&session_id=6994amigo1216798185&) |
| **MAPK10** | mitogen-activated protein kinase 10 | GO:0007165 : [signal transduction](http://amigo.geneontology.org/cgi-bin/amigo/term-details.cgi?term=GO:0007165&session_id=1435amigo1216798221&) |
| **ADORA2A** | Adenosine A2a receptor | GO:0008015 : [blood circulation](http://amigo.geneontology.org/cgi-bin/amigo/term-details.cgi?term=GO:0008015&session_id=9942amigo1216798250&)  GO:0007596 : [blood coagulation](http://amigo.geneontology.org/cgi-bin/amigo/term-details.cgi?term=GO:0007596&session_id=9942amigo1216798250&)  GO:0007267 : [cell-cell signaling](http://amigo.geneontology.org/cgi-bin/amigo/term-details.cgi?term=GO:0007267&session_id=9942amigo1216798250&)  GO:0006968 : [cellular defense response](http://amigo.geneontology.org/cgi-bin/amigo/term-details.cgi?term=GO:0006968&session_id=9942amigo1216798250&)  GO:0006954 : [inflammatory response](http://amigo.geneontology.org/cgi-bin/amigo/term-details.cgi?term=GO:0006954&session_id=9942amigo1216798250&)  GO:0006909 : [phagocytosis](http://amigo.geneontology.org/cgi-bin/amigo/term-details.cgi?term=GO:0006909&session_id=9942amigo1216798250&)  GO:0007600 : [sensory perception](http://amigo.geneontology.org/cgi-bin/amigo/term-details.cgi?term=GO:0007600&session_id=9942amigo1216798250&) |
| **SCCE** | kallikrein 7 (chymotryptic, stratum corneum) | GO:0008544 : [epidermis development](http://amigo.geneontology.org/cgi-bin/amigo/term-details.cgi?term=GO:0008544&session_id=8156amigo1216798362&) |
| **ADORA3** | Adenosine A3 receptor | GO:0006954 : [inflammatory response](http://amigo.geneontology.org/cgi-bin/amigo/term-details.cgi?term=GO:0006954&session_id=8616amigo1216798394&)  GO:0007165 : [signal transduction](http://amigo.geneontology.org/cgi-bin/amigo/term-details.cgi?term=GO:0007165&session_id=8616amigo1216798394&) |
| **NFKB1** | Nuclear factor of kappa light polypeptide gene enhancer in B-cells 1 (p105) | GO:0006954 : [inflammatory response](http://amigo.geneontology.org/cgi-bin/amigo/term-details.cgi?term=GO:0006954&session_id=4915amigo1216798435&)  GO:0045941 : [positive regulation of transcription](http://amigo.geneontology.org/cgi-bin/amigo/term-details.cgi?term=GO:0045941&session_id=4915amigo1216798435&)  GO:0006366 : [transcription from RNA polymerase II promoter](http://amigo.geneontology.org/cgi-bin/amigo/term-details.cgi?term=GO:0006366&session_id=4915amigo1216798435&) |
| **CCL18** | Chemokine (C-C motif) ligand 18 (pulmonary and activation-regulated) | GO:0007267 : [cell-cell signaling](http://amigo.geneontology.org/cgi-bin/amigo/term-details.cgi?term=GO:0007267&session_id=9360amigo1216798483&)  GO:0006955 : [immune response](http://amigo.geneontology.org/cgi-bin/amigo/term-details.cgi?term=GO:0006955&session_id=9360amigo1216798483&)  GO:0009607 : [response to biotic stimulus](http://amigo.geneontology.org/cgi-bin/amigo/term-details.cgi?term=GO:0009607&session_id=9360amigo1216798483&)  GO:0007165 : [signal transduction](http://amigo.geneontology.org/cgi-bin/amigo/term-details.cgi?term=GO:0007165&session_id=9360amigo1216798483&) |
| **AMBP** | Alpha-1-microglobulin/bikunin precursor | GO:0007155 : [cell adhesion](http://amigo.geneontology.org/cgi-bin/amigo/term-details.cgi?term=GO:0007155&session_id=3502amigo1216798532&)  GO:0050777 : [negative regulation of immune response](http://amigo.geneontology.org/cgi-bin/amigo/term-details.cgi?term=GO:0050777&session_id=3502amigo1216798532&) |
| **TACR1** | Tachykinin receptor 1 | GO:0009582 : [detection of abiotic stimulus](http://amigo.geneontology.org/cgi-bin/amigo/term-details.cgi?term=GO:0009582&session_id=4837amigo1216798591&)  GO:0006954 : [inflammatory response](http://amigo.geneontology.org/cgi-bin/amigo/term-details.cgi?term=GO:0006954&session_id=4837amigo1216798591&)  GO:0007638 : [mechanosensory behavior](http://amigo.geneontology.org/cgi-bin/amigo/term-details.cgi?term=GO:0007638&session_id=4837amigo1216798591&) |
| **KNG** | kininogen | GO:0006954 : [inflammatory response](http://amigo.geneontology.org/cgi-bin/amigo/term-details.cgi?term=GO:0006954&session_id=9992amigo1216798677&)  GO:0030195 : [negative regulation of blood coagulation](http://amigo.geneontology.org/cgi-bin/amigo/term-details.cgi?term=GO:0030195&session_id=9992amigo1216798677&)  GO:0007162 : [negative regulation of cell adhesion](http://amigo.geneontology.org/cgi-bin/amigo/term-details.cgi?term=GO:0007162&session_id=9992amigo1216798677&)  GO:0006939 : [smooth muscle contraction](http://amigo.geneontology.org/cgi-bin/amigo/term-details.cgi?term=GO:0006939&session_id=9992amigo1216798677&) |
| **BLNK** | B-cell linker | GO:0030183 : [B cell differentiation](http://amigo.geneontology.org/cgi-bin/amigo/term-details.cgi?term=GO:0030183&session_id=8881amigo1216798786&)  GO:0006959 : [humoral immune response](http://amigo.geneontology.org/cgi-bin/amigo/term-details.cgi?term=GO:0006959&session_id=8881amigo1216798786&)  GO:0006954 : [inflammatory response](http://amigo.geneontology.org/cgi-bin/amigo/term-details.cgi?term=GO:0006954&session_id=8881amigo1216798786&)  GO:0007242 : [intracellular signaling cascade](http://amigo.geneontology.org/cgi-bin/amigo/term-details.cgi?term=GO:0007242&session_id=8881amigo1216798786&) |
| **ABCF1** | ATP-binding cassette, sub-family F (GCN20), member 1 | GO:0006954 : [inflammatory response](http://amigo.geneontology.org/cgi-bin/amigo/term-details.cgi?term=GO:0006954&session_id=4390amigo1216798828&)  GO:0006412 : [translation](http://amigo.geneontology.org/cgi-bin/amigo/term-details.cgi?term=GO:0006412&session_id=4390amigo1216798828&) |
| **HPSE** | heparanase |  |
| **TLR7** | toll-like receptor 7 | GO:0051607 : [defense response to virus](http://amigo.geneontology.org/cgi-bin/amigo/term-details.cgi?term=GO:0051607&session_id=8402amigo1216798907&)  GO:0007249 : [I-kappaB kinase/NF-kappaB cascade](http://amigo.geneontology.org/cgi-bin/amigo/term-details.cgi?term=GO:0007249&session_id=8402amigo1216798907&)  GO:0045416 : [positive regulation of interleukin-8 biosynthetic process](http://amigo.geneontology.org/cgi-bin/amigo/term-details.cgi?term=GO:0045416&session_id=8402amigo1216798907&) |
| **IL22** | interleukin 22 | GO:0006953 : [acute-phase response](http://amigo.geneontology.org/cgi-bin/amigo/term-details.cgi?term=GO:0006953&session_id=4901amigo1216798972&)  GO:0007267 : [cell-cell signaling](http://amigo.geneontology.org/cgi-bin/amigo/term-details.cgi?term=GO:0007267&session_id=4901amigo1216798972&)  GO:0006954 : [inflammatory response](http://amigo.geneontology.org/cgi-bin/amigo/term-details.cgi?term=GO:0006954&session_id=4901amigo1216798972&) |
| **GPR132** | G protein-coupled receptor 132 | GO:0007186 : [G-protein coupled receptor protein signaling pathway](http://amigo.geneontology.org/cgi-bin/amigo/term-details.cgi?term=GO:0007186&session_id=439amigo1216799073&) |
| **IL1R** | Interleukin-1 receptor antagonist protein precursor | GO:0006955 : [immune response](http://amigo.geneontology.org/cgi-bin/amigo/term-details.cgi?term=GO:0006955&session_id=716amigo1216799111&)  GO:0006954 : [inflammatory response](http://amigo.geneontology.org/cgi-bin/amigo/term-details.cgi?term=GO:0006954&session_id=716amigo1216799111&) |
| **TOLLIP** | Toll interacting protein | GO:0007267 : [cell-cell signaling](http://amigo.geneontology.org/cgi-bin/amigo/term-details.cgi?term=GO:0007267&session_id=2660amigo1216799311&)  GO:0006954 : [inflammatory response](http://amigo.geneontology.org/cgi-bin/amigo/term-details.cgi?term=GO:0006954&session_id=2660amigo1216799311&)  GO:0007242 : [intracellular signaling cascade](http://amigo.geneontology.org/cgi-bin/amigo/term-details.cgi?term=GO:0007242&session_id=2660amigo1216799311&)  GO:0045321 : [leukocyte activation](http://amigo.geneontology.org/cgi-bin/amigo/term-details.cgi?term=GO:0045321&session_id=2660amigo1216799311&) |
| **IL8** | interleukin 8 | GO:0007267 : [cell-cell signaling](http://amigo.geneontology.org/cgi-bin/amigo/term-details.cgi?term=GO:0007267&session_id=8827amigo1216799375&)  GO:0007186 : [G-protein coupled receptor protein signaling pathway](http://amigo.geneontology.org/cgi-bin/amigo/term-details.cgi?term=GO:0007186&session_id=8827amigo1216799375&)  GO:0006954 : [inflammatory response](http://amigo.geneontology.org/cgi-bin/amigo/term-details.cgi?term=GO:0006954&session_id=8827amigo1216799375&)  GO:0007242 : [intracellular signaling cascade](http://amigo.geneontology.org/cgi-bin/amigo/term-details.cgi?term=GO:0007242&session_id=8827amigo1216799375&)  GO:0030155 : [regulation of cell adhesion](http://amigo.geneontology.org/cgi-bin/amigo/term-details.cgi?term=GO:0030155&session_id=8827amigo1216799375&)  GO:0045091 : [regulation of retroviral genome replication](http://amigo.geneontology.org/cgi-bin/amigo/term-details.cgi?term=GO:0045091&session_id=8827amigo1216799375&) |
| **TNFR** | CD27 antigen precursor | GO:0016064 : [immunoglobulin mediated immune response](http://amigo.geneontology.org/cgi-bin/amigo/term-details.cgi?term=GO:0016064&session_id=1994amigo1216799470&)  GO:0045579 : [positive regulation of B cell differentiation](http://amigo.geneontology.org/cgi-bin/amigo/term-details.cgi?term=GO:0045579&session_id=1994amigo1216799470&)  GO:0008588 : [release of cytoplasmic sequestered NF-kappaB](http://amigo.geneontology.org/cgi-bin/amigo/term-details.cgi?term=GO:0008588&session_id=1994amigo1216799470&) |
