# Supplementary material for: A systems biology approach to construct the gene regulatory network of systemic inflammation via microarray and databases mining
Source: BMC Med Genomics. 2008 Sep 30;1:46. doi: 10.1186/1755-8794-1-46 (PMC2567339; doi:10.1186/1755-8794-1-46)
Supplement: Additional file 2 — Supplementary Table 2. The inflammatory genes and their regulators [file 1755-8794-1-46-S2.doc]

**Supplementary Table 2:**

**The inflammatory genes and their regulators**

| Gene Name | (A)  Possible regulators from  JASPAR | (B) Candidate regulators from  Cross-correlation threshold | (C) Refined regulators from AIC |
| --- | --- | --- | --- |
| **IL17** | RUNX1,SOX9,RORA,MEF2A,HLF,TFAP2A,NFIL3,ELK1,FOXD1,FOXL1,GATA2,FOXI1,IRF1,YY1,REL,RELA,NFKB1,SPIB | RUNX1,SOX9,RORA,HLF,TFAP2A,NFIL3,ELK1,FOXD1,FOXL1,GATA2,FOXI1,YY1,REL,RELA,NFKB1,SPIB | RUNX1,SOX9,RORA,HLF,TFAP2A,NFIL3,ELK1,FOXD1,GATA2,FOXI1,YY1,REL,RELA,NFKB1,SPIB |
| **IL1A** | RUNX1,Pbx,SOX9,MEF2A,HLF,TFAP2A,E2F1,NFIL3,ELK1,FOXD1,FOXL1,GATA2,FOXI1,YY1,REL,RELA,NFKB1,SPIB | RUNX1,Pbx,SOX9,HLF,TFAP2A,E2F1,NFIL3,ELK1,FOXD1,FOXL1,GATA2,FOXI1,YY1,REL,RELA,NFKB1 | RUNX1,Pbx,HLF,TFAP2A,NFIL3,ELK1,FOXD1,FOXL1,GATA2,FOXI1,YY1,REL,RELA,NFKB1 |
| **TNFA** | TFAP2A,FOXD1,FOXL1,GATA2,FOXI1,MAX,YY1,NFKB1,SPIB | TFAP2A,FOXD1,FOXL1,GATA2,FOXI1,MAX,YY1,NFKB1,SPIB | TFAP2A,GATA2,FOXI1,MAX,YY1,NFKB1,SPIB |
| **IL6** | RUNX1,MEF2A,HLF,TFAP2A,ELK1,FOXL1,GATA2,FOXI1,YY1,REL,RELA,NFKB1,SPIB | RUNX1,HLF,TFAP2A,ELK1,FOXL1,GATA2,FOXI1,YY1,REL,NFKB1 | RUNX1,HLF,TFAP2A,FOXL1,GATA2,FOXI1,YY1,REL,NFKB1 |
| **IL1B** | SOX9,MEF2A,TFAP2A,ELK1,FOXD1,FOXL1,GATA2,FOXI1,YY1,REL,RELA,NFKB1,SPIB | SOX9,MEF2A,ELK1,GATA2,FOXI1,REL,RELA,NFKB1,SPIB | SOX9,MEF2A,ELK1,GATA2,FOXI1,RELA,NFKB1 |
| **TLR4** | RUNX1,Pbx,SOX9,RORA,MEF2A,HLF,TFAP2A,NFIL3,ELK1,FOXF2,FOXD1,FOXL1,GATA2,FOXI1,IRF1,MAX,YY1,REL,RELA,SPIB | RUNX1,MEF2A,NFIL3,ELK1,FOXI1,IRF1,MAX,REL,RELA,SPIB | RUNX1,MEF2A,FOXI1,IRF1,MAX,REL,RELA,SPIB |
| **NFATC3** | SOX9,HLF,TFAP2A,NFIL3,ELK1,FOXD1,FOXL1,GATA2,FOXI1,MAX,YY1,REL | SOX9,HLF,NFIL3,ELK1,FOXD1,GATA2,FOXI1,MAX,YY1 | SOX9,HLF,NFIL3,ELK1,FOXD1,GATA2,FOXI1,MAX |
| **SCYE1** | RUNX1,SOX9,TFAP2A,FOXD1,FOXL1,GATA2,FOXI1,YY1,REL,SPIB | RUNX1,TFAP2A,FOXL1,REL,SPIB | RUNX1,TFAP2A,FOXL1,REL,SPIB |
| **TICAM2** | SOX9,RORA,TFAP2A,ELK1,FOXL1,GATA2,YY1,REL,RELA,SPIB | SOX9,TFAP2A,ELK1,FOXL1,REL,RELA,SPIB | SOX9,TFAP2A,ELK1,FOXL1,RELA,SPIB |
| **HDAC4** | SOX9,HLF,TFAP2A,E2F1,NFIL3,ELK1,FOXL1,GATA2,YY1,REL | TFAP2A,FOXL1 | TFAP2A,FOXL1 |
| **HDAC5** | RUNX1,SOX9,HLF,TFAP2A,NFIL3,ELK1,FOXD1,FOXL1,GATA2,FOXI1,MAX,YY1,REL,SPIB | RUNX1,FOXD1,FOXL1,FOXI1,MAX,YY1,REL,SPIB | RUNX1,FOXD1,FOXL1,MAX,YY1,REL,SPIB |
| **HDAC7A** | RUNX1,SOX9,TFAP2A,E2F1,ELK1,FOXD1,FOXL1,GATA2,FOXI1,YY1,NFKB1,SPIB | SOX9,TFAP2A,E2F1,FOXD1,FOXL1,GATA2,FOXI1,YY1,NFKB1,SPIB | TFAP2A,E2F1,FOXD1,FOXL1,GATA2,FOXI1,YY1,NFKB1,SPIB |
| **HDAC9** | SOX9,RORA,MEF2A,TFAP2A,NFIL3,ELK1,FOXD1,FOXL1,GATA2,FOXI1,IRF1,YY1,REL,SPIB | MEF2A,NFIL3,IRF1,REL,SPIB | NFIL3,IRF1,REL |
| **ITGB2** | RUNX1,SOX9,TFAP2A,ELK1,FOXD1,FOXL1,GATA2,FOXI1,IRF1,MAX,YY1,SPIB | RUNX1,SOX9,TFAP2A,ELK1,FOXD1,FOXL1,GATA2,FOXI1,IRF1,MAX,YY1,SPIB | RUNX1,TFAP2A,ELK1,FOXD1,FOXL1,GATA2,FOXI1,IRF1,MAX,YY1,SPIB |
| **CXCL2** | RUNX1,Pbx,SOX9,RORA,E2F1,NFIL3,FOXF2,FOXD1,FOXL1,GATA2,FOXI1,IRF1,YY1,SPIB | RUNX1,Pbx,SOX9,RORA,E2F1,NFIL3,FOXF2,FOXD1,FOXL1,GATA2,FOXI1,IRF1,YY1 | SOX9,RORA,E2F1,NFIL3,FOXD1,FOXL1,GATA2 |
| **ALOX5** | RUNX1,Pbx,SOX9,RORA,MEF2A,TFAP2A,FOXD1,FOXL1,GATA2,FOXI1,MAX,YY1,SPIB | RUNX1,MEF2A,TFAP2A,FOXL1,SPIB | RUNX1,MEF2A,TFAP2A,SPIB |
| **NFKBIA** | RUNX1,SOX9,MEF2A,TFAP2A,FOXD1,FOXL1,GATA2,FOXI1,IRF1,YY1,SPIB | RUNX1,SOX9,MEF2A,GATA2,FOXI1,IRF1,SPIB | RUNX1,MEF2A,FOXI1,IRF1,SPIB |
| **NR3C1** | RUNX1,Pbx,SOX9,RORA,MEF2A,TFAP2A,FOXD1,FOXL1,GATA2,FOXI1,MAX,YY1,SPIB | RUNX1,Pbx,SOX9,RORA,MEF2A,TFAP2A,FOXD1,FOXL1,GATA2,FOXI1,YY1,SPIB | RUNX1,Pbx,SOX9,MEF2A,TFAP2A,FOXD1,FOXL1,GATA2,FOXI1,YY1,SPIB |
| **CEBPD** | TFAP2A,NFIL3,FOXF2,FOXD1,FOXL1,GATA2,FOXI1,YY1,REL,SPIB | NFIL3,FOXF2,FOXI1,REL,SPIB | NFIL3,FOXF2,FOXI1,SPIB |
| **ANXA1** | GATA2,YY1,SPIB | SPIB | SPIB |
| **CYBB** | RUNX1,SOX9,TFAP2A,FOXD1,FOXL1,GATA2,FOXI1,IRF1,YY1,REL,SPIB | FOXL1,IRF1,SPIB | FOXL1,SPIB |
| **AOAH** | RUNX1,SOX9,RORA,HLF,TFAP2A,ELK1,FOXF2,FOXD1,FOXL1,GATA2,FOXI1,YY1,REL,RELA,SPIB | RUNX1,TFAP2A,ELK1,FOXL1,FOXI1,RELA,SPIB | RUNX1,FOXL1,FOXI1,RELA |
| **REG3A** | SOX9,RORA,ELK1,FOXL1,GATA2,FOXI1,YY1,SPIB | SOX9,RORA,ELK1,FOXL1,GATA2,FOXI1,YY1,SPIB | SOX9,RORA,ELK1,FOXL1,GATA2,SPIB |
| **FOS** | RUNX1,SOX9,TFAP2A,E2F1,ELK1,FOXD1,FOXL1,GATA2,FOXI1,YY1,SPIB | RUNX1,TFAP2A,ELK1,FOXL1 | RUNX1,TFAP2A,ELK1,FOXL1 |
| **IRAK** | RUNX1,SOX9,RORA,TFAP2A,E2F1,ELK1,FOXD1,FOXL1,GATA2,MAX,YY1,SPIB | E2F1,MAX,SPIB | E2F1,SPIB |
| **PLAA** | RUNX1,Pbx,SOX9,RORA,MEF2A,HLF,TFAP2A,NFIL3,ELK1,FOXF2,FOXD1,FOXL1,GATA2,FOXI1,IRF1,MAX,YY1,SPIB | RUNX1,Pbx,SOX9,RORA,HLF,TFAP2A,ELK1,FOXF2,FOXD1,FOXL1,GATA2,FOXI1,MAX,YY1,SPIB | RUNX1,SOX9,TFAP2A,FOXF2,FOXD1,FOXL1,GATA2,MAX,SPIB |
| **CCR7** | RUNX1,SOX9,TFAP2A,ELK1,FOXD1,GATA2,IRF1,YY1,REL,SPIB | IRF1,REL,SPIB | IRF1,REL |
| **CXCL14** | RUNX1,SOX9,RORA,HLF,TFAP2A,E2F1,ELK1,FOXD1,FOXL1,GATA2,FOXI1,IRF1,YY1,REL,RELA,SPIB | RUNX1,SOX9,RORA,HLF,TFAP2A,E2F1,ELK1,FOXD1,FOXL1,GATA2,FOXI1,YY1,REL,RELA,SPIB | RUNX1,SOX9,HLF,TFAP2A,E2F1,FOXD1,FOXL1,GATA2,YY1,REL,RELA,SPIB |
| **PLA2G4B** | MEF2A,TFAP2A,E2F1,NFIL3,ELK1,FOXL1,GATA2,FOXI1,YY1,REL,SPIB | MEF2A,E2F1,NFIL3,GATA2,FOXI1,YY1,SPIB | MEF2A,NFIL3,FOXI1,YY1,SPIB |
| **NFRKB** | RUNX1,SOX9,RORA,MEF2A,TFAP2A,E2F1,FOXD1,FOXL1,GATA2,FOXI1,MAX,YY1,REL,SPIB | RUNX1,SOX9,RORA,E2F1,FOXD1,GATA2,FOXI1,MAX,YY1,REL,SPIB | RUNX1,SOX9,RORA,E2F1,FOXD1,REL,SPIB,GATA2,FOXI1,MAX,YY1 |
| **MAPK10** | RUNX1,SOX9,MEF2A,HLF,TFAP2A,E2F1,ELK1,FOXL1GATA2,FOXI1,IRF1,YY1,REL,SPIB | RUNX1,SOX9,HLF,TFAP2A,E2F1,ELK1,FOXL1,GATA2FOXI1,YY1,REL,SPIB | RUNX1,SOX9,TFAP2A,E2F1,ELK1,FOXL1,GATA2,FOXI1,REL,SPIB |
| **ADORA2A** | RUNX1,SOX9,RORA,TFAP2A,E2F1,FOXD1,FOXL1,GATA2,IRF1,YY1,REL,RELA,SPIB | RUNX1,SOX9,RORA,TFAP2A,E2F1,FOXD1,FOXL1,GATA2,IRF1,YY1,REL,RELA,SPIB | RUNX1,RORA,TFAP2A,E2F1,,REL,RELA,SPIB,FOXL1,GATA2,IRF1,YY1 |
| **SCCE** | RUNX1,Pbx,SOX9,RORA,HLF,TFAP2A,ELK1,FOXD1,FOXL1,GATA2,FOXI1,IRF1,MAX,YY1,SPIB | RUNX1,Pbx,SOX9,RORA,HLF,TFAP2A,ELK1,FOXD1,FOXL1,GATA2,FOXI1,MAX,YY1,SPIB | RUNX1,SOX9,RORA,HLF,ELK1,FOXD1,FOXL1,GATA2,YY1,SPIB |
| **ADORA3** | RUNX1,Pbx,ELK1,FOXL1,GATA2,YY1 | Pbx,GATA2 | Pbx,GATA2 |
| **NFKB1** | RUNX1,SOX9,TFAP2A,ELK1,FOXD1,FOXL1,GATA2,FOXI1,YY1,REL,SPIB | RUNX1,SOX9,ELK1,GATA2,FOXI1,YY1,REL,SPIB | RUNX1,SOX9,ELK1,GATA2,FOXI1,YY1,SPIB |
| **CCL18** | Pbx,SOX9,TFAP2A,NFIL3,FOXD1,FOXL1,GATA2,YY1,SPIB | Pbx,SOX9,TFAP2A,NFIL3,FOXD1,FOXL1,GATA2,YY1 | Pbx,SOX9,TFAP2A,NFIL3,FOXL1,GATA2,YY1 |
| **AMBP** | RUNX1,TFAP2A,ELK1,FOXF2,FOXD1,FOXL1,GATA2,FOXI1,MAX,YY1,REL,SPIB | RUNX1,TFAP2A,ELK1,FOXF2,FOXD1,FOXL1,GATA2,FOXI1,MAX,YY1,REL,SPIB | TFAP2A,FOXF2,FOXD1,FOXL1,GATA2,FOXI1,MAX,YY1,REL,SPIB |
| **TACR1** | RUNX1,SOX9,RORA,TFAP2A,E2F1,FOXD1,FOXL1,GATA2,MAX,YY1,SPIB | RUNX1,SOX9,RORA,TFAP2A,E2F1,FOXD1,FOXL1,GATA2,MAX,YY1,SPIB | RUNX1,SOX9,RORA,TFAP2A,E2F1,FOXD1,FOXL1,MAX,YY1,SPIB |
| **KNG** | RUNX1,SOX9,RORA,TFAP2A,FOXL1,GATA2,FOXI1,YY1,REL,SPIB | RUNX1,SOX9,RORA,TFAP2A,FOXL1,GATA2,FOXI1,YY1,REL,SPIB | RUNX1,SOX9,TFAP2A,FOXL1,GATA2,FOXI1,YY1,REL |
| **BLNK** | SOX9,MEF2A,NFIL3,ELK1,FOXF2,FOXD1,FOXL1,GATA2,FOXI1,YY1,SPIB | MEF2A,NFIL3,FOXI1,SPIB | MEF2A,FOXI1 |
| **ABCF1** | RUNX1,SOX9,RORA,TFAP2A,E2F1,ELK1,FOXD1,FOXL1,GATA2,FOXI1,MAX,YY1,REL,RELA,NFKB1,SPIB | RUNX1,SOX9,E2F1,ELK1,GATA2,FOXI1,MAX,YY1,REL,RELA,NFKB1,SPIB | RUNX1,E2F1,ELK1,GATA2,FOXI1,MAX,YY1,REL,RELA,NFKB1 |
| **HPSE** | RUNX1,SOX9,TFAP2A,E2F1,ELK1,FOXD1,FOXL1,GATA2,FOXI1,YY1,REL,SPIB | REL,SPIB | REL |
| **TLR7** | RUNX1,SOX9,MEF2A,HLFTFAP2A,FOXD1,FOXL1,GATA2,FOXI1,IRF1,YY1,REL,RELA,NFKB1,SPIB | RUNX1,MEF2A,IRF1,REL,RELA,NFKB1,SPIB | RUNX1,MEF2A,IRF1,REL,NFKB1,SPIB |
| **IL22** | RUNX1,SOX9,RORA,MEF2A,TFAP2A,E2F1,ELK1,FOXD1,FOXL1,GATA2,FOXI1,MAX,YY1,REL,NFKB1,SPIB | RUNX1,SOX9,RORA,TFAP2A,E2F1,ELK1,FOXD1,FOXL1,GATA2,FOXI1,MAX,YY1,REL,NFKB1,SPIB | RUNX1,SOX9,RORA,E2F1,MAX,YY1,REL,NFKB1,SPIB,ELK1,FOXD1,FOXL1,GATA2,FOXI1 |
| **GPR132** | SOX9,RORA,MEF2A,TFAP2A,E2F1,FOXL1,GATA2,FOXI1,IRF1,YY1,NFKB1,SPIB | SOX9,RORA,MEF2A,TFAP2A,E2F1,FOXL1,GATA2,FOXI1,IRF1,YY1,NFKB1,SPIB | SOX9,RORA,MEF2A,TFAP2A,E2F1,FOXL1,GATA2,FOXI1,IRF1,YY1,SPIB |
| **IL1R** | RUNX1,SOX9,HLF,TFAP2A,NFIL3,ELK1,FOXD1,FOXL1,GATA2,FOXI1,YY1,REL,RELA,SPIB | TFAP2A,NFIL3,FOXL1,RELA,SPIB | TFAP2A,NFIL3,FOXL1,RELA |
| **TOLLIP** | RUNX1,SOX9,RORA,MEF2A,HLF,TFAP2A,NFIL3,ELK1,FOXD1,FOXL1,GATA2,FOXI1,MAX,YY1,REL,RELA,NFKB1,SPIB | RUNX1,SOX9,RORA,HLF,TFAP2A,NFIL3,ELK1,YY1,REL,RELA,NFKB1,SPIB,FOXD1,FOXL1,GATA2,FOXI1,MAX | RUNX1,SOX9,RORA,HLF,TFAP2A,NFIL3,ELK1,YY1,REL,RELA,NFKB1,FOXD1,FOXL1,GATA2,FOXI1,MAX |
| **IL8** | RUNX1,Pbx,SOX9,RORA,MEF2A,HLF,TFAP2A,E2F1,NFIL3,ELK1,FOXF2,FOXD1,FOXL1,GATA2,FOXI1,MAX,YY1,REL,RELA,SPIB | RUNX1,Pbx,SOX9,RORA,MEF2A,HLF,E2F1,NFIL3,ELK1,FOXF2,FOXD1,GATA2,FOXI1,MAX,YY1,REL,RELA | RUNX1,SOX9,RORA,MEF2A,HLF,E2F1,NFIL3,ELK1,FOXF2,FOXD1,GATA2,FOXI1,MAX,YY1,REL,RELA |
| **TNFR** | Pbx,SOX9,TFAP2A,FOXL1,GATA2,FOXI1 | FOXI1 | FOXI1 |
