# Supplementary material for: A systems biology approach to construct the gene regulatory network of systemic inflammation via microarray and databases mining
Source: BMC Med Genomics. 2008 Sep 30;1:46. doi: 10.1186/1755-8794-1-46 (PMC2567339; doi:10.1186/1755-8794-1-46)
Supplement: Additional file 3 — Supplementary Table 3. The gene regulatory network in immune system of un-activated and inflammatory cells [file 1755-8794-1-46-S3.doc]

**Supplementary Table 3:**

**The gene regulatory network in immune system of un-activated and inflammatory cells**

|  | **In un-activated Condition** | **In Inflammatory Condition** |
| --- | --- | --- |
| **IL17** | RUNX1,SOX9,RORA,MEF2A,HLF,TFAP2A,NFIL3,  ELK1,FOXD1,FOXL1,FOXI1,IRF1,YY1,REL,NFKB1,SPIB | RUNX1,SOX9,RORA,HLF,TFAP2A,NFIL3,ELK1,FOXD1,  GATA2,FOXI1,YY1,REL,RELA,NFKB1,SPIB |
| **IL1A** | RUNX1,Pbx,SOX9,MEF2A,HLF,TFAP2A,E2F1,  NFIL3,ELK1,FOXD1,FOXL1,FOXI1,YY1,REL | RUNX1,Pbx,HLF,TFAP2A,NFIL3,ELK1,FOXD1,FOXL1,  GATA2,FOXI1,YY1,REL,RELA,NFKB1 |
| **TNFa** | TFAP2A,FOXD1,GATA2,FOXI1,MAX,NFKB1 | TFAP2A,GATA2,FOXI1,MAX,YY1,NFKB1,SPIB |
| **IL6** | RUNX1,MEF2A,HLF,FOXL1,GATA2,FOXI1,YY1,  REL,RELA,SPIB | RUNX1,HLF,TFAP2A,FOXL1,GATA2,FOXI1,YY1,REL,  NFKB1 |
| **IL1B** | SOX9,MEF2A,TFAP2A,ELK1,FOXD1,FOXL1,  GATA2,FOXI1,YY1,REL,NFKB1,SPIB | SOX9,MEF2A,ELK1,GATA2,FOXI1,RELA,NFKB1 |
| **TLR4** | RUNX1,Pbx,SOX9,RORA,MEF2A,HLF,TFAP2A,  NFIL3,ELK1,FOXF2,FOXD1,FOXL1,GATA2,FOXI1,IRF1,MAX,YY1,REL,RELA,SPIB | RUNX1,MEF2A,FOXI1,IRF1,MAX,REL,RELA,SPIB |
| **NFATC3** | NFIL3,ELK1,FOXD1,FOXI1,MAX,YY1,REL | SOX9,HLF,NFIL3,ELK1,FOXD1,GATA2,FOXI1,MAX |
| **SCYE1** | RUNX1,FOXI1,YY1,SPIB | RUNX1,TFAP2A,FOXL1,REL,SPIB |
| **TICAM2** | RORA,GATA2,YY1,REL,RELA | SOX9,TFAP2A,ELK1,FOXL1,RELA,SPIB |
| **HDAC4** | YY1 | TFAP2A,FOXL1 |
| **HDAC5** | NFIL3,FOXI1,YY1,REL,SPIB | RUNX1,FOXD1,FOXL1,MAX,YY1,REL,SPIB |
| **HDAC7A** | RUNX1,ELK1,FOXD1,GATA2,YY1,NFKB1,SPIB | TFAP2A,E2F1,FOXD1,FOXL1,GATA2,FOXI1,YY1  NFKB1,SPIB, |
| **HDAC9** | NFIL3,SPIB | NFIL3,IRF1,REL |
| **ITGB2** | RUNX1,ELK1,FOXD1,GATA2,FOXI1,IRF1,MAX | RUNX1,TFAP2A,ELK1,FOXD1,FOXL1,GATA2,FOXI1,  IRF1,MAX,YY1,SPIB |
| **CXCL2** | RUNX1,RORA,E2F1,NFIL3,FOXF2,GATA2,FOXI1,  IRF | SOX9,RORA,E2F1,NFIL3,FOXD1,FOXL1,GATA2 |
| **ALOX5** | RORA,FOXD1,YY1,SPIB | RUNX1,MEF2A,TFAP2A,SPIB |
| **NFKBIA** | MEF2A,FOXI1 | RUNX1,MEF2A,FOXI1,IRF1,SPIB |
| **NR3C1** | RORA,FOXD1,FOXI1,YY1 | RUNX1,Pbx,SOX9,MEF2A,TFAP2A,FOXD1,FOXL1,  GATA2,FOXI1,YY1,SPIB |
| **CEBPD** | TFAP2A,FOXF2,FOXD1,FOXL1,GATA2,FOXI1,  YY1,REL | NFIL3,FOXF2,FOXI1,SPIB |
| **ANXA1** | YY1,SPIB | SPIB |
| **CYBB** | FOXI1,IRF1,YY1,REL,SPIB | FOXL1,SPIB |
| **AOAH** | RORA,TFAP2A,FOXF2,FOXD1,FOXL1,YY1,REL  ,RELA,SPIB | RUNX1,FOXL1,FOXI1,RELA |
| **REG3A** | RORA,FOXI1,YY1,SPIB | SOX9,RORA,ELK1,FOXL1,GATA2,SPIB |
| **FOS** | SOX9,E2F1,GATA2,FOXI1,YY1,SPIB | RUNX1,TFAP2A,ELK1,FOXL1 |
| **IRAK** | RUNX1,SOX9,RORA,E2F1,FOXD1,GATA2,MAX,  YY1,SPIB | E2F1,SPIB |
| **PLAA** | RUNX1,MEF2A,FOXF2,FOXD1,GATA2,FOXI1,  IRF1,MAX,YY1,SPIB | RUNX1,SOX9,TFAP2A,FOXF2,FOXD1,FOXL1,  GATA2,MAX,SPIB |
| **CCR7** | IRF1,SPIB | IRF1,REL |
| **CXCL14** | GATA2,FOXI1,IRF1,YY1,REL,RELA,SPIB | RUNX1,SOX9,HLF,TFAP2A,E2F1,FOXD1,FOXL1,  GATA2,YY1,REL,RELA,SPIB |
| **PLA2G4B** | E2F1,FOXI1,YY1,REL,SPIB | MEF2A,NFIL3,FOXI1,YY1,SPIB |
| **NFRKB** | RORA,FOXD1,FOXI1,SPIB | RUNX1,SOX9,RORA,E2F1,FOXD1,REL,SPIB,GATA2,  FOXI1,MAX,YY1 |
| **MAPK10** | E2F1,IRF1,REL,SPIB | RUNX1,SOX9,TFAP2A,E2F1,ELK1,FOXL1,GATA2,  FOXI1,REL,SPIB |
| **ADORA2A** | RUNX1,RORA,FOXD1,GATA2,IRF1,YY1,REL,  RELA,SPIB | RUNX1,RORA,TFAP2A,E2F1,,REL,RELA,SPIB,FOXL1,  GATA2,IRF1,YY1 |
| **SCCE** | RUNX1,FOXD1,SPIB | RUNX1,SOX9,RORA,HLF,ELK1,FOXD1,FOXL1,GATA2,  YY1,SPIB |
| **ADORA3** | RUNX1,GATA2,YY1 | Pbx,GATA2 |
| **NFKB1** | RUNX1,ELK1,REL,SPIB | RUNX1,SOX9,ELK1,GATA2,FOXI1,YY1,SPIB |
| **CCL18** | FOXD1,GATA2,YY1,SPIB | Pbx,SOX9,TFAP2A,NFIL3,FOXL1,GATA2,YY1 |
| **AMBP** | FOXF2,FOXD1,FOXI1,MAX,YY1,REL,SPIB | TFAP2A,FOXF2,FOXD1,FOXL1,GATA2,FOXI1,MAX,  YY1,REL,SPIB |
| **TACR1** | RUNX1,E2F1,FOXD1,YY1 | RUNX1,SOX9,RORA,TFAP2A,E2F1,FOXD1,FOXL1,  MAX,YY1,SPIB |
| **KNG** | RUNX1,FOXI1,YY1,REL,SPIB | RUNX1,SOX9,TFAP2A,FOXL1,GATA2,FOXI1,YY1,REL |
| **BLNK** | NFIL3 | MEF2A,FOXI1 |
| **ABCF1** | RUNX1,E2F1,FOXD1,YY1,RELA,NFKB1,SPIB | RUNX1,E2F1,ELK1,GATA2,FOXI1,MAX,YY1,REL,RELA,  NFKB1 |
| **HPSE** | E2F1,ELK1,FOXD1,FOXI1,YY1,REL,SPIB, | REL |
| **TLR7** | RUNX1,MEF2A,FOXD1,YY1,REL,SPIB | RUNX1,MEF2A,IRF1,REL,NFKB1,SPIB |
| **IL22** | RUNX1,SOX9,E2F1,FOXD1,GATA2,FOXI1,REL,  NFKB1,SPIB | RUNX1,SOX9,RORA,E2F1,MAX,YY1,REL,NFKB1,SPIB,  ELK1,FOXD1,FOXL1,GATA2,FOXI1 |
| **GPR132** | E2F1,GATA2,IRF1,NFKB1,SPIB | SOX9,RORA,MEF2A,TFAP2A,E2F1,FOXL1,GATA2,  FOXI1,IRF1,YY1,SPIB |
| **IL1R** | RUNX1,SOX9,HLF,TFAP2A,NFIL3,ELK1,FOXD1,  GATA2,YY1,REL,RELA,SPIB | TFAP2A,NFIL3,FOXL1,RELA |
| **TOLLIP** | ELK1,FOXD1,GATA2,FOXI1,MAX,YY1,RELA,  NFKB1,SPIB | RUNX1,SOX9,RORA,HLF,TFAP2A,NFIL3,ELK1,  YY1,REL,RELA,NFKB1,FOXD1,FOXL1,GATA2,  FOXI1,MAX |
| **IL8** | Pbx,SOX9,RORA,MEF2A,HLF,TFAP2A,E2F1,  NFIL3,ELK1,FOXF2,FOXD1,FOXL1,GATA2,FOXI1,MAX,YY1,REL,SPIB | RUNX1,SOX9,RORA,MEF2A,HLF,E2F1,NFIL3,ELK1,  FOXF2,FOXD1,GATA2,FOXI1,MAX,YY1,REL,RELA |
| **TNFR** | Pbx,SOX9,TFAP2A,FOXL1,GATA2,FOXI1 | FOXI1 |
