# Supplementary material for: A systems biology approach to construct the gene regulatory network of systemic inflammation via microarray and databases mining
Source: BMC Med Genomics. 2008 Sep 30;1:46. doi: 10.1186/1755-8794-1-46 (PMC2567339; doi:10.1186/1755-8794-1-46)
Supplement: Additional file 4 — Supplementary Table 4. Gene Connectivities only in inflammatory condition but not in normal condition [file 1755-8794-1-46-S4.doc]

**Supplementary Table 4:**

**Gene Connectivities only in inflammatory condition but not in normal condition**

| Screen Name | Orf Name | Description | goFunction | goProcess | Connectivities |
| --- | --- | --- | --- | --- | --- |
| **FOXL1** | HGNC:3817 | forkhead box L1 | sequence-specific DNA binding;DNA bending activity;transcription factor activity; | regulation of transcription, DNA-dependent;multicellular organismal development;transcription; | 23 |
| **TFAP2A** | HGNC:11742 | transcription factor AP-2 alpha (activating enhancer binding protein 2 alpha) | protein dimerization activity;protein binding;transcription coactivator activity;RNA polymerase II transcription factor activity, enhancer binding;transcription factor activity; | regulation of transcription from RNA polymerase II promoter;signal transduction;regulation of transcription, DNA-dependent;ectoderm development;transcription; | 19 |
| **SOX9** | HGNC:11204 | SRY (sex determining region Y)-box 9 (campomelic dysplasia, autosomal sex-reversal) | DNA binding;specific RNA polymerase II transcription factor activity;protein binding;transcriptional activator activity; | regulation of apoptosis;positive regulation of transcription from RNA polymerase II promoter;regulation of transcription from RNA polymerase II promoter;male germ-line sex determination;regulation of transcription, DNA-dependent;transcription;negative regulation of transcription, DNA-dependent;hair follicle development;epithelial to mesenchymal transition;regulation of cell proliferation;male gonad development;heart development;neural crest cell development;cell fate specification;cartilage condensation;skeletal development; | 16 |
| **GATA2** | HGNC:4171 | GATA binding protein 2 | zinc ion binding;metal ion binding;sequence-specific DNA binding;transcription factor activity; | neuron differentiation;phagocytosis;regulation of transcription, DNA-dependent;transcription;pituitary gland development;positive regulation of phagocytosis;transcription from RNA polymerase II promoter;cell maturation; | 12 |
| **AML1** | HGNC:10471 | runt-related transcription factor 1 (acute myeloid leukemia 1; aml1 oncogene) | molecular_function;ATP binding;molecular function unknown;transcription factor activity;protein binding;chloride ion binding;transcriptional activator activity; | positive regulation of transcription from RNA polymerase II promoter;regulation of transcription, DNA-dependent;transcription;behavioral response to pain;hemopoiesis;multicellular organismal development;biological_process;neuron development;positive regulation of granulocyte differentiation;positive regulation of angiogenesis;biological process unknown;skeletal development; | 11 |
| **NR3C1** | HGNC:7978 | nuclear receptor subfamily 3, group C, member 1 (glucocorticoid receptor) | DNA binding;steroid hormone receptor activity;ligand-dependent nuclear receptor activity;sequence-specific DNA binding;transcription factor activity;receptor activity;zinc ion binding;glucocorticoid receptor activity;protein binding;metal ion binding;lipid binding;steroid binding; | regulation of transcription, DNA-dependent;transcription;transcription from RNA polymerase II promoter;inflammatory response;signal transduction;sex determination; | 8 |
| **YY1** | HGNC:12856 | YY1 transcription factor | zinc ion binding;metal ion binding;protein binding;transcription corepressor activity;transcription coactivator activity;transcription factor activity; | regulation of transcription from RNA polymerase II promoter;eye morphogenesis (sensu Mammalia);transcription;antimicrobial humoral response (sensu Vertebrata);anterior/posterior pattern formation; | 7 |
| **SCCE** | HGNC:6368 | kallikrein-related peptidase 7 | trypsin activity;serine-type endopeptidase activity;chymotrypsin activity; | epidermis development;proteolysis; | 7 |
| **GPR132** | HGNC:17482 | G protein-coupled receptor 132 | rhodopsin-like receptor activity;receptor activity; | signal transduction;G-protein coupled receptor protein signaling pathway;G1/S transition of mitotic cell cycle; | 7 |
| **CXCL14** | HGNC:10640 | chemokine (C-X-C motif) ligand 14 | chemokine activity; | signal transduction;chemotaxis;cell-cell signaling;immune response;inflammatory response; | 7 |
| **TOLLIP** | HGNC:16476 | toll interacting protein | Toll binding;signal transducer activity;protein binding; | leukocyte activation;intracellular signaling cascade;cell-cell signaling;immune response;phosphorylation;inflammatory response; | 7 |
| **NFKB1** | HGNC:7794 | nuclear factor of kappa light polypeptide gene enhancer in B-cells 1 (p105) | specific transcriptional repressor activity;protein binding;transcription factor activity; | signal transduction;regulation of transcription, DNA-dependent;negative regulation of interleukin-12 biosynthetic process;regulation of transcription;response to pathogenic bacteria;inflammatory response;negative regulation of transcription, DNA-dependent;positive regulation of transcription;antibacterial humoral response (sensu Vertebrata);transcription from RNA polymerase II promoter;anti-apoptosis;apoptosis; | 7 |
| **SPIB** | HGNC:11242 | Spi-B transcription factor (Spi-1/PU.1 related) | sequence-specific DNA binding;RNA polymerase II transcription factor activity;transcription factor activity; | regulation of transcription from RNA polymerase II promoter;macrophage differentiation;transcription; | 7 |
| **NFRKB** | HGNC:7802 | nuclear factor related to kappaB binding protein | specific RNA polymerase II transcription factor activity;DNA binding; | inflammatory response;transcription from RNA polymerase II promoter; | 7 |
| **MAPK10** | HGNC:6872 | mitogen-activated protein kinase 10 | ATP binding;protein-tyrosine kinase activity;MAP kinase kinase activity;MAP kinase activity;protein binding;JUN kinase activity;transferase activity;nucleotide binding;protein serine/threonine kinase activity;protein kinase activity; | signal transduction;JNK cascade;protein amino acid phosphorylation; | 7 |
| **FOXI1** | HGNC:3815 | forkhead box I1 | transcriptional activator activity;sequence-specific DNA binding;DNA bending activity;transcription factor activity; | multicellular organismal development;regulation of transcription, DNA-dependent;sensory perception of sound;inner ear morphogenesis;transcription;positive regulation of transcription, DNA-dependent; | 7 |
| **ELK-1** | ELK-1 | user_defined_node | NONE | NONE | 7 |
| **MAX** | HGNC:6913 | MYC associated factor X | protein binding;transcription coactivator activity;transcription regulator activity;transcription factor activity;DNA binding; | regulation of transcription, DNA-dependent;regulation of transcription;transcription;transcription from RNA polymerase II promoter; | 6 |
| **TACR1** | HGNC:11526 | tachykinin receptor 1 | receptor activity;tachykinin receptor activity;rhodopsin-like receptor activity; | tachykinin signaling pathway;mechanosensory behavior;signal transduction;G-protein coupled receptor protein signaling pathway;inflammatory response;response to pain;G-protein signaling, coupled to IP3 second messenger (phospholipase C activating);detection of abiotic stimulus; | 6 |
| **C-REL** | HGNC:9954 | v-rel reticuloendotheliosis viral oncogene homolog (avian) | signal transducer activity;protein binding;transcription factor activity; | positive regulation of I-kappaB kinase/NF-kappaB cascade;regulation of transcription, DNA-dependent;positive regulation of interleukin-12 biosynthetic process;positive regulation of transcription, DNA-dependent;transcription from RNA polymerase II promoter;cytokine production; | 6 |
| **TICAM2** | HGNC:21354 | toll-like receptor adaptor molecule 2 | protein binding;signal transducer activity;transmembrane receptor activity;protein carrier activity; | positive regulation of I-kappaB kinase/NF-kappaB cascade;intracellular protein transport;inflammatory response;transport; | 5 |
| **CCL18** | HGNC:10616 | chemokine (C-C motif) ligand 18 (pulmonary and activation-regulated) | cytokine activity;chemokine activity; | signal transduction;chemotaxis;cell-cell signaling;immune response;sensory perception;inflammatory response;antimicrobial humoral response (sensu Vertebrata);response to biotic stimulus; | 5 |
| **ABCF1** | HGNC:70 | ATP-binding cassette, sub-family F (GCN20), member 1 | ATP binding;ATPase activity;nucleoside-triphosphatase activity;ATPase activity, coupled to transmembrane movement of substances;nucleotide binding;iron ion binding;electron transporter activity;iron-sulfur cluster binding;unfolded protein binding;translation factor activity, nucleic acid binding;metal ion binding;electron carrier activity;heat shock protein binding; | translation;inflammatory response;electron transport;transport;protein folding; | 5 |
| **IL22** | HGNC:14900 | interleukin 22 | interleukin-22 receptor binding;cytokine activity; | immune response;inflammatory response;acute-phase response;cell-cell signaling; | 5 |
| **RORA** | HGNC:10258 | RAR-related orphan receptor A | zinc ion binding;metal ion binding;protein binding;steroid hormone receptor activity;sequence-specific DNA binding;transcription factor activity; | signal transduction;regulation of transcription, DNA-dependent;transcription;cGMP metabolic process;regulation of macrophage activation;nitric oxide biosynthetic process; | 5 |
| **MEF2A** | HGNC:6993 | MADS box transcription enhancer factor 2, polypeptide A (myocyte enhancer factor 2A) | protein binding;transcription coactivator activity;sequence-specific DNA binding;transcription factor activity;DNA binding; | regulation of transcription, DNA-dependent;transcription;muscle development;positive regulation of transcription;transcription from RNA polymerase II promoter; | 5 |
| **KNG** | HGNC:6383 | kininogen 1 | zinc ion binding;protein binding;cysteine protease inhibitor activity;heparin binding;receptor binding; | smooth muscle contraction;natriuresis;diuresis;vasodilation;negative regulation of cell adhesion;positive regulation of apoptosis;negative regulation of blood coagulation;inflammatory response;blood pressure regulation;blood coagulation; | 4 |
| **FOS** | HGNC:3796 | v-fos FBJ murine osteosarcoma viral oncogene homolog | protein dimerization activity;sequence-specific DNA binding;specific RNA polymerase II transcription factor activity;transcription factor activity;DNA binding; | regulation of transcription from RNA polymerase II promoter;regulation of transcription, DNA-dependent;nervous system development;inflammatory response;DNA methylation; | 4 |
| **REG3A** | HGNC:8601 | regenerating islet-derived 3 alpha | sugar binding; | multicellular organismal development;heterophilic cell adhesion;cell proliferation;inflammatory response;acute-phase response; | 4 |
| **HDAC7A** | HGNC:14067 | histone deacetylase 7A | specific transcriptional repressor activity;transcription factor binding;transcription corepressor activity;histone deacetylase activity;hydrolase activity; | negative regulation of transcription from RNA polymerase II promoter;regulation of transcription, DNA-dependent;nervous system development;transcription;negative regulation of striated muscle development;inflammatory response;regulation of progression through cell cycle;chromatin modification;B cell differentiation; | 4 |
| **RELA** | HGNC:9955 | v-rel reticuloendotheliosis viral oncogene homolog A, nuclear factor of kappa light polypeptide gene enhancer in B-cells 3, p65 (avian) | phosphate binding;RNA polymerase II transcription factor activity, enhancer binding;protein kinase activity;transcription factor activity;NF-kappaB binding;signal transducer activity;protein kinase binding;protein binding;identical protein binding;protein N-terminus binding; | defense response to virus;anti-apoptosis;liver development;regulation of transcription, DNA-dependent;negative regulation of protein catabolic process;response to organic substance;regulation of transcription;positive regulation of transcription, DNA-dependent;cytokine and chemokine mediated signaling pathway;hair follicle development;positive regulation of interleukin-12 biosynthetic process;transcription from RNA polymerase II promoter;inflammatory response;response to toxin;positive regulation of I-kappaB kinase/NF-kappaB cascade;cellular defense response;activation of NF-kappaB transcription factor;response to UV-B; | 4 |
| **E2F1** | HGNC:3113 | E2F transcription factor 1 | protein binding;transcription corepressor activity;transcription factor activity; | negative regulation of transcription from RNA polymerase II promoter;regulation of transcription, DNA-dependent;G1 phase of mitotic cell cycle;transcription;cell cycle;cell proliferation;regulation of progression through cell cycle;apoptosis; | 4 |
| **NFIL3** | HGNC:7787 | nuclear factor, interleukin 3 regulated | protein dimerization activity;transcription corepressor activity;sequence-specific DNA binding;transcription factor activity;DNA binding; | regulation of transcription, DNA-dependent;immune response;transcription from RNA polymerase II promoter; | 4 |
| **HDAC5** | HGNC:14068 | histone deacetylase 5 | catalytic activity;transcription corepressor activity;hydrolase activity;histone deacetylase activity;transcription factor binding;specific transcriptional repressor activity; | negative regulation of transcription from RNA polymerase II promoter;regulation of transcription, DNA-dependent;transcription;chromatin remodeling;inflammatory response;negative regulation of striated muscle development;regulation of progression through cell cycle;heart development;B cell differentiation;chromatin modification;chromatin silencing; | 4 |
| **ITGB2** | HGNC:6155 | integrin, beta 2 (complement component 3 receptor 3 and 4 subunit) | receptor activity;protein kinase binding;protein binding; | cell-cell signaling;apoptosis;leukocyte adhesion;cell adhesion;neutrophil chemotaxis;antimicrobial humoral response (sensu Vertebrata);multicellular organismal development;inflammatory response;regulation of peptidyl-tyrosine phosphorylation;integrin-mediated signaling pathway;cell-matrix adhesion;regulation of cell shape; | 4 |
| **HLF1** | HLF1 | user_defined_node | NONE | NONE | 4 |
| **CXCL2** | HGNC:4603 | chemokine (C-X-C motif) ligand 2 | cytokine activity;chemokine activity; | chemotaxis;G-protein coupled receptor protein signaling pathway;immune response;sensory perception;inflammatory response;response to stimulus; | 3 |
| **IL1A** | HGNC:5991 | interleukin 1, alpha | interleukin-1 receptor binding;signal transducer activity;protein binding; | fever;chemotaxis;cell-cell signaling;negative regulation of cell proliferation;immune response;cell proliferation;inflammatory response;regulation of progression through cell cycle;anti-apoptosis;apoptosis; | 3 |
| **IRF1** | HGNC:6116 | interferon regulatory factor 1 | transcription factor activity; | regulation of transcription, DNA-dependent;positive regulation of interleukin-12 biosynthetic process;transcription;cell cycle;immune response;positive regulation of transcription, DNA-dependent;negative regulation of progression through cell cycle;transcription from RNA polymerase II promoter; | 3 |
| **PBX1** | HGNC:8632 | pre-B-cell leukemia transcription factor 1 | DNA binding;sequence-specific DNA binding;transcription factor activity;protein heterodimerization activity;protein binding; | sex differentiation;ureteric bud branching;urogenital system development;positive regulation of transcription from RNA polymerase II promoter;regulation of transcriptional preinitiation complex formation;regulation of transcription, DNA-dependent;embryonic development;organ morphogenesis;hindbrain development;transcription from RNA polymerase II promoter;cell differentiation;adrenal gland development;C21-steroid hormone biosynthetic process;spleen development;positive regulation of cell proliferation; | 3 |
| **NFATC3** | HGNC:7777 | nuclear factor of activated T-cells, cytoplasmic, calcineurin-dependent 3 | protein binding;transcription coactivator activity;transcription factor activity; | heart development;regulation of transcription from RNA polymerase II promoter;regulation of transcription, DNA-dependent;inflammatory response;cellular respiration; | 3 |
| **NFKBIA** | HGNC:7797 | nuclear factor of kappa light polypeptide gene enhancer in B-cells inhibitor, alpha | transcription factor binding;NF-kappaB binding;nuclear localization sequence binding;ubiquitin protein ligase binding; | protein import into nucleus, translocation;regulation of NF-kappaB import into nucleus;negative regulation of Notch signaling pathway;negative regulation of myeloid cell differentiation;negative regulation of DNA binding;regulation of cell proliferation;response to pathogenic bacteria;cytoplasmic sequestering of NF-kappaB;apoptosis; | 3 |
| **SCYE1** | HGNC:10648 | small inducible cytokine subfamily E, member 1 (endothelial monocyte-activating) | cytokine activity;tRNA binding;nucleic acid binding; | signal transduction;chemotaxis;cell-cell signaling;inflammatory response;tRNA aminoacylation for protein translation;translation; | 3 |
| **PLAA** | HGNC:9043 | phospholipase A2-activating protein | protein binding;phospholipase A2 activator activity;binding; | signal transduction;phospholipid metabolic process;inflammatory response; | 3 |
| **ADORA2A** | HGNC:263 | adenosine A2a receptor | gastric inhibitory peptide receptor activity;adenosine receptor activity, G-protein coupled;receptor activity;signal transducer activity;rhodopsin-like receptor activity;G-protein coupled receptor activity;A3 adenosine receptor activity, G-protein coupled;A2A adenosine receptor activity, G-protein coupled; | cell-cell signaling;synaptic transmission, dopaminergic;apoptosis;G-protein signaling, coupled to cAMP nucleotide second messenger;G-protein coupled receptor protein signaling pathway;neurotransmitter transport;sensory perception;circulation;adenosine receptor signaling pathway;central nervous system development;inflammatory response;signal transduction;adenylate cyclase activation;phagocytosis;blood coagulation;cellular defense response;cAMP biosynthetic process;eating behavior;locomotory behavior; | 3 |
| **FOXD1** | HGNC:3802 | forkhead box D1 | sequence-specific DNA binding;DNA bending activity;transcription factor activity; | regulation of transcription, DNA-dependent;transcription; | 3 |
| **ALOX5** | RP11-67C2.3 | arachidonate 5-lipoxygenase | lipoxygenase activity;arachidonate 5-lipoxygenase activity;protein binding;calcium ion binding;iron ion binding;oxidoreductase activity; | electron transport;inflammatory response;leukotriene metabolic process;leukotriene biosynthetic process; | 3 |
| **AMBP** | HGNC:453 | alpha-1-microglobulin/bikunin precursor | IgA binding;trypsin inhibitor activity;transporter activity;plasmin inhibitor activity;calcium channel inhibitor activity;heme binding;protein homodimerization activity;calcium oxalate binding;serine-type endopeptidase inhibitor activity; | negative regulation of JNK cascade;cell adhesion;protein-chromophore linkage;anti-inflammatory response;transport;pregnancy;negative regulation of immune response;heme catabolic process; | 3 |
| **TNFA** | DASS-280D8.2 | tumor necrosis factor (TNF superfamily, member 2) | protein binding;cytokine activity;tumor necrosis factor receptor binding; | leukocyte adhesion;anti-apoptosis;apoptosis;organ morphogenesis;negative regulation of glucose import;positive regulation of I-kappaB kinase/NF-kappaB cascade;regulation of immunoglobulin secretion;multicellular organismal development;regulation of protein amino acid phosphorylation;glucose metabolic process;regulation of transcription, DNA-dependent;positive regulation of transcription from RNA polymerase II promoter;defense response to bacterium;positive regulation of transcription;negative regulation of transcription from RNA polymerase II promoter;cell-cell signaling;response to virus;cell death;response to wounding;humoral immune response;immune response;signal transduction;inflammatory response;regulation of cell proliferation;positive regulation of translational initiation by iron;regulation of osteoclast differentiation;induction of apoptosis via death domain receptors;protein import into nucleus, translocation;positive regulation of JNK cascade;activation of NF-kappaB transcription factor;cellular ex | 3 |
| **HDAC4** | HGNC:14063 | histone deacetylase 4 | DNA binding;hydrolase activity;histone deacetylase activity;transcription factor binding;transcriptional repressor activity; | negative regulation of transcription from RNA polymerase II promoter;regulation of transcription, DNA-dependent;cell cycle;transcription;multicellular organismal development;inflammatory response;negative regulation of striated muscle development;nervous system development;B cell differentiation;chromatin modification;skeletal development;negative regulation of cell proliferation; | 2 |
| **BLNK** | HGNC:14211 | B-cell linker | protein binding;transmembrane receptor protein tyrosine kinase adaptor protein activity;SH3/SH2 adaptor activity; | intracellular signaling cascade;humoral immune response;inflammatory response;hemocyte development (sensu Arthropoda);B cell activation;B cell differentiation; | 2 |
| **TLR7** | HGNC:15631 | toll-like receptor 7 | siRNA binding;protein binding;transmembrane receptor activity;single-stranded RNA binding;double-stranded RNA binding; | defense response to virus;positive regulation of interferon-gamma biosynthetic process;immune response;positive regulation of interleukin-8 biosynthetic process;inflammatory response;positive regulation of interferon-beta biosynthetic process;positive regulation of interferon-alpha biosynthetic process; | 2 |
| **AOAH** | HGNC:548 | acyloxyacyl hydrolase (neutrophil) | catalytic activity;acyloxyacyl hydrolase activity;lipoprotein lipase activity;hydrolase activity, acting on ester bonds;hydrolase activity; | lipopolysaccharide metabolic process;inflammatory response;lipid metabolic process;negative regulation of inflammatory response; | 2 |
| **IL6** | HGNC:6018 | interleukin 6 (interferon, beta 2) | cytokine activity;protein binding;interleukin-6 receptor binding; | neuron differentiation;cell surface receptor linked signal transduction;cell-cell signaling;humoral immune response;negative regulation of apoptosis;negative regulation of chemokine biosynthetic process;negative regulation of cell proliferation;positive regulation of cell proliferation;neutrophil apoptosis;immune response;acute-phase response;B cell differentiation; | 2 |
| **IL8** | HGNC:6025 | interleukin 8 | chemokine activity;protein binding;cytokine activity;interleukin-8 receptor binding; | cell-cell signaling;response to stimulus;neutrophil activation;calcium-mediated signaling;G-protein coupled receptor protein signaling pathway;neutrophil chemotaxis;sensory perception;cell motility;immune response;intracellular signaling cascade;inflammatory response;angiogenesis;regulation of cell adhesion;induction of positive chemotaxis;cell cycle arrest;chemotaxis;regulation of retroviral genome replication;negative regulation of cell proliferation; | 2 |
| **CEBPD** | HGNC:1835 | CCAAT/enhancer binding protein (C/EBP), delta | sequence-specific DNA binding;transcription factor activity;protein dimerization activity;DNA binding; | regulation of transcription, DNA-dependent;transcription from RNA polymerase II promoter;transcription; | 2 |
| **PLA2G4B** | HGNC:9036 | phospholipase A2, group IVB (cytosolic) | calcium-dependent phospholipase A2 activity;calcium-dependent phospholipid binding;calcium ion binding;lysophospholipase activity;phospholipase activity;hydrolase activity; | phospholipid catabolic process;glycerophospholipid catabolic process;calcium-mediated signaling;inflammatory response;arachidonic acid metabolic process;lipid catabolic process;parturition; | 2 |
| **IL17** | HGNC:5981 | interleukin 17A | cytokine activity; | cell-cell signaling;protein amino acid glycosylation;immune response;inflammatory response;cell death;apoptosis; | 2 |
| **HDAC9** | HGNC:14065 | histone deacetylase 9 | hydrolase activity;transcription corepressor activity;histone deacetylase activity;transcription factor binding;specific transcriptional repressor activity; | negative regulation of transcription from RNA polymerase II promoter;regulation of transcription, DNA-dependent;histone deacetylation;transcription;inflammatory response;negative regulation of striated muscle development;regulation of progression through cell cycle;heart development;B cell differentiation;chromatin modification; | 2 |
| **IL1B** | HGNC:5992 | interleukin 1, beta | signal transducer activity;interleukin-1 receptor binding;protein binding;growth factor activity;interleukin-1 receptor antagonist activity; | cell-cell signaling;apoptosis;neutrophil chemotaxis;antimicrobial humoral response (sensu Vertebrata);positive regulation of interleukin-6 biosynthetic process;immune response;inflammatory response;fever;signal transduction;positive regulation of chemokine biosynthetic process;regulation of progression through cell cycle;leukocyte migration;negative regulation of cell proliferation;cell proliferation; | 1 |
| **IL1R** | HGNC:5993 | interleukin 1 receptor, type I | receptor activity;protein binding;interleukin-1, Type I, activating receptor activity;interleukin-1 receptor activity;transmembrane receptor activity; | cell surface receptor linked signal transduction;cytokine and chemokine mediated signaling pathway;immune response;inflammatory response; | 1 |
| **CYBB** | HGNC:2578 | cytochrome b-245, beta polypeptide (chronic granulomatous disease) | metal ion binding;electron transporter activity;iron ion binding;voltage-gated ion channel activity;FAD binding;oxidoreductase activity; | electron transport;inflammatory response;antimicrobial humoral response (sensu Vertebrata);ion transport; | 1 |
| **CCR7** | HGNC:1608 | chemokine (C-C motif) receptor 7 | receptor activity;C-C chemokine receptor activity;rhodopsin-like receptor activity; | signal transduction;chemotaxis;G-protein coupled receptor protein signaling pathway;elevation of cytosolic calcium ion concentration;inflammatory response;antimicrobial humoral response (sensu Vertebrata); | 1 |
| **ADORA3** | HGNC:268 | adenosine A3 receptor | receptor activity;A3 adenosine receptor activity, G-protein coupled;rhodopsin-like receptor activity; | signal transduction;adenylate cyclase activation;regulation of heart contraction;G-protein coupled receptor protein signaling pathway;inflammatory response; | 1 |
| **TNFR1** | HGNC:11916 | tumor necrosis factor receptor superfamily, member 1A | receptor activity;protein binding;enzyme binding;tumor necrosis factor receptor activity; | cytokine and chemokine mediated signaling pathway;signal transduction;positive regulation of I-kappaB kinase/NF-kappaB cascade;inflammatory response;prostaglandin metabolic process;positive regulation of transcription from RNA polymerase II promoter;positive regulation of inflammatory response;apoptosis; | 0 |
| **ANXA1** | HGNC:533 | annexin A1 | protein binding, bridging;structural molecule activity;receptor binding;calcium ion binding;phospholipase inhibitor activity;calcium-dependent phospholipid binding;phospholipase A2 inhibitor activity; | anti-apoptosis;peptide cross-linking;cell cycle;cell motility;regulation of cell proliferation;inflammatory response;cell surface receptor linked signal transduction;arachidonic acid secretion;lipid metabolic process;keratinocyte differentiation; | 0 |
| **IRAK** | HGNC:6112 | interleukin-1 receptor-associated kinase 1 | ATP binding;protein serine/threonine kinase activity;protein kinase activity;nucleotide binding;receptor activity;transferase activity;NF-kappaB-inducing kinase activity;interleukin-1 receptor binding;magnesium ion binding;protein kinase binding;protein homodimerization activity;protein binding;kinase activity;transcriptional activator activity; | protein amino acid autophosphorylation;positive regulation of transcription;cytokine and chemokine mediated signaling pathway;protein oligomerization;defense response;signal transduction;transmembrane receptor protein serine/threonine kinase signaling pathway;activation of NF-kappaB-inducing kinase;protein amino acid phosphorylation; | 0 |
| **HPSE** | HGNC:5164 | heparanase | hydrolase activity;beta-glucuronidase activity;calcium ion binding;magnesium ion binding; | inflammatory response;proteoglycan metabolic process; | 0 |
| **FOXF2** | HGNC:3810 | forkhead box F2 | transcription coactivator activity;sequence-specific DNA binding;RNA polymerase II transcription factor activity;transcription factor activity; | establishment of epithelial cell polarity;embryonic gut development;regulation of transcription, DNA-dependent;extracellular matrix organization and biogenesis;organ morphogenesis;lung development;transcription;vasculogenesis;positive regulation of transcription, DNA-dependent;transcription from RNA polymerase II promoter; | 0 |
| **TLR4** | HGNC:11850 | toll-like receptor 4 | receptor activity;transmembrane receptor activity;protein binding;lipopolysaccharide binding; | detection of pathogenic bacteria;macrophage activation;detection of fungus;positive regulation of interleukin-13 biosynthetic process;positive regulation of interleukin-6 biosynthetic process;innate immune response;positive regulation of interleukin-12 biosynthetic process;positive regulation of interleukin-1 biosynthetic process;inflammatory response;negative regulation of osteoclast differentiation;signal transduction;positive regulation of JNK cascade;positive regulation of I-kappaB kinase/NF-kappaB cascade;response to bacterium;activation of NF-kappaB-inducing kinase;mast cell activation;T-helper 1 type immune response; | 0 |
