# Supplementary material for: A systems biology approach to construct the gene regulatory network of systemic inflammation via microarray and databases mining
Source: BMC Med Genomics. 2008 Sep 30;1:46. doi: 10.1186/1755-8794-1-46 (PMC2567339; doi:10.1186/1755-8794-1-46)
Supplement: Additional file 5 — Supplementary Material S1–S9. Identification of time delay in Step 7 [file 1755-8794-1-46-S5.doc]

**Supplementary Material S1-S9:**

**Identification of time delay in Step 7**

Supplementary S1

$$\begin{matrix} X_{IL1A}[n+1]=a_{1}X_{IL1A}[n]+a_{2}X_{RUNX1}[n]+a_{3}X_{PBX}[n]+a_{4}X_{HLF}[n] \\ \text{ }+a_{5}X_{TFAP2A}[n]+a_{6}X_{C-REL}[n]+a_{7}X_{NFIL3}[n]+a_{8}X_{ELK1}[n] \\ \text{ }+a_{9}X_{FOXD1}[n]+a_{10}X_{FOXL1}[n]+a_{11}X_{GATA2}[n]+a_{12}X_{FOXI1}[n] \\ \text{ +}a_{13}X_{\text{YY}1}[n]+a_{14}X_{RELA}[n]+a_{15}X_{NFKB1}[n] \end{matrix}$$

Supplementary S2

$$\begin{matrix} X_{IL1B}[n+1]=b_{1}X_{IL1B}[n]+b_{2}X_{SOX9}[n]+b_{3}X_{MEF2A}[n-3]+b_{4}X_{ELK1}[n] \\ +b_{5}X_{FOXI1}[n]+b_{6}X_{GATA2}[n]+b_{7}X_{NFKB1}[n]+b_{8}X_{RELA}[n] \\ \end{matrix}$$

Supplementary S3

$$\begin{matrix} X_{IL1R}[n+1]=c_{1}X_{IL1R}[n]+c_{2}X_{TFAP2A}[n]+c_{3}X_{NFIL3}[n] +c_{4}X_{FOXL1}[n] \\ +c_{5}X_{RELA}[n-4] \\ \end{matrix}$$

Supplementary S4

$$\begin{matrix} X_{IL6}[n+1]=e_{1}X_{IL6}[n]+e_{2}X_{RUNX1}[n]+e_{3}X_{HLF}[n]+e_{4}X_{TFAP2A}[n] \\ +e_{5}X_{FOXL2}[n]+e_{6}X_{GATA2}[n]+e_{7}X_{FOXI1}[n] \\ \text{ }+e_{8}X_{YY1}[n] +e_{9}X_{REL}[n]+e_{10}X_{NFKB1}[n] \\ \end{matrix}$$

Supplementary S5

$$\begin{matrix} X_{IL8}[n+1]=i_{1}X_{IL8}[n]+i_{2}X_{PBX}[n]+i_{3}X_{SOX9}[n-1]+i_{4}X_{RORA}[n] \\ \text{ }+i_{5}X_{MEF2A}[n-2]+i_{6}X_{HLF}[n]+i_{7}X_{E2F1}[n]+i_{8}X_{NFIL3}[n-1] \\ \text{ }+i_{9}X_{ELK1}[n-1]+i_{10}X_{FOXF1}[n-1]+i_{11}X_{FOXD1}[n-1]+i_{12}X_{GATA2}[n-1] \\ \text{ }+i_{13}X_{FOXI1}[n-1]+i_{14}X_{MAX}[n]+i_{15}X_{YY1}[n]+i_{16}X_{REL}[n-1] \\ +i_{17}X_{RELA}[n-1] \end{matrix}$$

Supplementary S6

$$\begin{matrix} X_{IL17}[n+1]=f_{1}X_{IL17}[n]+f_{2}X_{RUNX1}[n]+f_{3}X_{RORA}[n]+f_{4}X_{HLF}[n] \\ \text{ }+f_{5}X_{TFAP2A}[n]+f_{6}X_{NFIL3}[n]+f_{7}X_{ELK1}[n]+f_{8}X_{FOXD1}[n] \\ \text{ }+f_{9}X_{FOXL1}[n]+f_{10}X_{GATA2}[n]+f_{11}X_{FOXI1}[n]+f_{12}X_{YY1}[n-1] \\ \text{ }+f_{13}X_{C-REL}[n]+f_{14}X_{RELA}[n]+f_{15}X_{NFKB1}[n]+f_{16}X_{SPIB}[n-2] \end{matrix}$$

Supplementary S7

$$\begin{matrix} X_{TLR4}[n+1]=g_{1}X_{TLR4}[n]+g_{2}X_{RUNX1}[n-2]+g_{3}X_{MEF2A}[n]+g_{4}X_{FOXI1}[n] \\ +g_{5}X_{IRF1}[n]+g_{6}X_{MAX}[n-4]+g_{7}X_{C-REL}[n-4]+g_{8}X_{RELA}[n-1] \\ +g_{9}X_{SPIB}[n] \\ \end{matrix}$$

Supplementary S8

$$\begin{matrix} X_{TNFA}[n+1]=d_{1}X_{TNFA}[n]+d_{2}X_{TFAP2A}[n]+d_{3}X_{FOXD1}[n]+d_{4}X_{GATA2}[n] \\ +d_{5}X_{FOXI1}[n]+d_{6}X_{MAX}[n]+d_{7}X_{NFKB1}[n]+d_{8}X_{SPIB}[n-1] \\ \end{matrix}$$

Supplementary S9

$$X_{TNFR}[n+1]=h_{1}X_{TNFR}[n]+h_{2}X_{FOXI1}[n]$$
