# Supplementary material for: A systems biology approach to construct the gene regulatory network of systemic inflammation via microarray and databases mining
Source: BMC Med Genomics. 2008 Sep 30;1:46. doi: 10.1186/1755-8794-1-46 (PMC2567339; doi:10.1186/1755-8794-1-46)
Supplement: Additional file 6 — Supplementary Table 5. The parameters of the inflammatory gene regulator models for Additional file 1 [file 1755-8794-1-46-S6.doc]

**Supplementary Table 5:**

**The estimated parameter data for Additional file 1**

| **The parameters of the inflammatory gene regulator models for S1-S9** | | | | | | | | | | | | | | | | | |
| --- | --- | --- | --- | --- | --- | --- | --- | --- | --- | --- | --- | --- | --- | --- | --- | --- | --- |
| $a_{1}$ | 286.3 | $b_{1}$ | 1.142 | $c_{1}$ | 0.7432 | $d_{1}$ | 1.476 | $e_{1}$ | -3.185 | $f_{1}$ | -37.22 | $g_{1}$ | 1.026 | $h_{1}$ | 0.9369 | $i_{1}$ | -0.06601 |
| $a_{2}$ | -256.5 | $b_{2}$ | -7.899 | $c_{2}$ | 17.12 | $d_{2}$ | -7.888 | $e_{2}$ | -5.678 | $f_{2}$ | -92.1 | $g_{2}$ | 3.744 | $h_{2}$ | 1.019 | $i_{2}$ | 124.4 |
| $a_{3}$ | 2678 | $b_{3}$ | -6.776 | $c_{3}$ | -13.89 | $d_{3}$ | 18.35 | $e_{3}$ | 186.2 | $f_{3}$ | 157.6 | $g_{3}$ | -2.814 |  |  | $i_{3}$ | -601.6 |
| $a_{4}$ | -3107 | $b_{4}$ | 30.71 | $c_{4}$ | -6.779 | $d_{4}$ | -15.36 | $e_{4}$ | -59.94 | $f_{4}$ | -490 | $g_{4}$ | 10.97 |  |  | $i_{4}$ | 1113 |
| $a_{5}$ | -756.8 | $b_{5}$ | 7.043 | $c_{5}$ | 8.942 | $d_{5}$ | -1.185 | $e_{5}$ | -7.987 | $f_{5}$ | 310.2 | $g_{5}$ | 5.266 |  |  | $i_{5}$ | -431 |
| $a_{6}$ | -33.95 | $b_{6}$ | -10.74 |  |  | $d_{6}$ | 4.209 | $e_{6}$ | 94.24 | $f_{6}$ | 308.8 | $g_{6}$ | 5.693 |  |  | $i_{6}$ | 29.29 |
| $a_{7}$ | -1538 | $b_{7}$ | -32.87 |  |  | $d_{7}$ | -2.06 | $e_{7}$ | -164.2 | $f_{7}$ | -12.31 | $g_{7}$ | -3.598 |  |  | $i_{7}$ | -928.5 |
| $a_{8}$ | 571.5 | $b_{8}$ | 13.85 |  |  | $d_{8}$ | -3.281 | $e_{8}$ | 38.94 | $f_{8}$ | 164.1 | $g_{8}$ | -5.625 |  |  | $i_{8}$ | -378.9 |
| $a_{9}$ | -255.4 |  |  |  |  |  |  | $e_{9}$ | -32.11 | $f_{9}$ | -117.6 | $g_{9}$ | -13.6 |  |  | $i_{9}$ | -37.33 |
| $a_{10}$ | -1458 |  |  |  |  |  |  | $e_{10}$ | -0.4727 | $f_{10}$ | 95.22 |  |  |  |  | $i_{10}$ | 636.3 |
| $a_{11}$ | 312.5 |  |  |  |  |  |  |  |  | $f_{11}$ | 215.3 |  |  |  |  | $i_{11}$ | 708.3 |
| $a_{12}$ | 456 |  |  |  |  |  |  |  |  | $f_{12}$ | -6.024 |  |  |  |  | $i_{12}$ | -211.7 |
| $a_{13}$ | -98.25 |  |  |  |  |  |  |  |  | $f_{13}$ | -106.6 |  |  |  |  | $i_{13}$ | 226.4 |
| $a_{14}$ | -3.591 |  |  |  |  |  |  |  |  | $f_{14}$ | -4.48 |  |  |  |  | $i_{14}$ | 237.1 |
| $a_{15}$ | 4.879 |  |  |  |  |  |  |  |  | $f_{15}$ | 4.975 |  |  |  |  | $i_{15}$ | -260.6 |
|  |  |  |  |  |  |  |  |  |  | $f_{16}$ | 1.567 |  |  |  |  | $i_{16}$ | -268.5 |
|  |  |  |  |  |  |  |  |  |  |  |  |  |  |  |  | $i_{17}$ | 34.03 |

| **Standard deviations for each gene** | |
| --- | --- |
| L1A | 0.017 |
| IL1B | 0.012 |
| IL1R | 0.051 |
| IL6 | 0.0035 |
| IL8 | 0.0219 |
| IL17 | 0.011 |
| TNFA | 0.046 |
| TLR4 | 0.0059 |
| TNFR | 0.0842 |
