# Supplementary material for: A systems biology approach to construct the gene regulatory network of systemic inflammation via microarray and databases mining
Source: BMC Med Genomics. 2008 Sep 30;1:46. doi: 10.1186/1755-8794-1-46 (PMC2567339; doi:10.1186/1755-8794-1-46)
Supplement: Additional file 7 — Supplementary Material S10. Reconstruction via independent data [file 1755-8794-1-46-S7.doc]

**Supplementary Material S10:**

**Reconstruction via independent data**

**Dataset selection**

We used the microarray data of Boldrick et al. [38] as our mRNA expression profiles. Gene expression in whole blood leukocytes was determined at 0, 0.5, 1, 2, 4, 6, 12 and 24 h after the intravenous administration of bacterial endotoxin to Human peripheral blood mononuclear cells (PBMCs). In their experiments, eight additional stresses were studied under identical conditions, we adopt the MONK-treated sample as our normal condition and the LPS-treated sample as the inflammatory condition. The infusion of endotoxin activates innate immune responses and presents with physiological responses of brief duration. It should be noted that there is an initial proinflammatory phase and a subsequent counter regulatory phase, with resolution of virtually all clinical perturbation within 24 h.

**Network construction**

In the first step, we pick up 9 genes which are common induction and 10 genes of the host response which are common repression (See Table 1 and 2). These genes are discussed in the innate immune response from microarray of Boldrick et. al. (2002) [38] and we want to select the candidate regulators of these 19 target genes in diversity pathogen infect in innate immune response. We would construct the gene regulatory network in both inflammatory and normal cases. The section of regulators needs to analyze changes in blood leukocyte gene expression patterns by Temporal Relationship Identification Algorithm (TRIA) prediction algorithm [23]. The TRIA prediction algorithm could identify the regulators by the correlations among continuous time gene expressions with the assumption that the regulatory genes and target genes have a positively (negatively) temporal relationship if the target gene’s expression profile is positively (negatively) correlated with the regulatory genes profile, possibly with time lags.

We follow the steps of the flowchart in the main manuscript then rebuild the gene regulatory network in both inflammation condition and normal condition (see Fig 1 and 2). And the Fig. 3 shows the gene network only in inflammatory condition but not in normal condition. The results of the selection in inflammation condition are shown in Table3.

**Discussion**

After comparing the reconstructed inflammatory gene regulatory network with the one in the text, we found some similarities and differences. The same highly connected hubs are GATA2, AML1 (RUNX1) and YY1. There are more than 5 connections for these hubs in both perturbed inflammatory networks. However, for the lack of some specific gene expression data in reference [38], we were unable to verify a part of highly interactive genes in the text (i.e. FOXL1, TFAP2A and SOX9). Interestingly, we also found there are some hubs only present in the reconstructed network but not in the text like GATA3 and FPR, which would be involved in host defense against bacterial infection and in the clearance of damaged cells [39]. The reason why these 19 candidate genes still discovered new hubs is because some of 19 candidate genes are not included in the previous 49 genes. For different experimental conditions, research topics and technology platforms, the data pool from different literature may be different. Therefore, the candidates of target genes we chose here

differed from the text, so the computational results would not be identical.

**T**able 1: Features of the Host Response: Common Induction

| **Gene Symbol** | **Description** | **GO bio-Function** |
| --- | --- | --- |
| CD40 | TNF receptor superfamily member 5 | GO:0042100 : [B cell proliferation](http://amigo.geneontology.org/cgi-bin/amigo/term-details.cgi?term=GO:0042100&session_id=5606amigo1218010654&)  GO:0006954 : [inflammatory response](http://amigo.geneontology.org/cgi-bin/amigo/term-details.cgi?term=GO:0006954&session_id=5606amigo1218010654&)  GO:0030168 : [platelet activation](http://amigo.geneontology.org/cgi-bin/amigo/term-details.cgi?term=GO:0030168&session_id=5606amigo1218010654&)  GO:0043123 : [positive regulation of I-kappaB kinase/NF-kappaB cascade](http://amigo.geneontology.org/cgi-bin/amigo/term-details.cgi?term=GO:0043123&session_id=5606amigo1218010654&)  GO:0006461 : [protein complex assembly](http://amigo.geneontology.org/cgi-bin/amigo/term-details.cgi?term=GO:0006461&session_id=5606amigo1218010654&) |
| CSF | Combining with macrophage colony stimulating factor to initiate a change in cell activity. | GO:0008283 : [cell proliferation](http://amigo.geneontology.org/cgi-bin/amigo/term-details.cgi?term=GO:0008283&session_id=5555amigo1218010796&)  GO:0007275:[multicellular organismal development](http://amigo.geneontology.org/cgi-bin/amigo/term-details.cgi?term=GO:0007275&session_id=5555amigo1218010796&)  GO:0007165 : [signal transduction](http://amigo.geneontology.org/cgi-bin/amigo/term-details.cgi?term=GO:0007165&session_id=5555amigo1218010796&) |
| IL-1α | Interleukin-1 alpha precursor | GO:0006954 : [inflammatory response](http://amigo.geneontology.org/cgi-bin/amigo/term-details.cgi?term=GO:0006954&session_id=4384amigo1216796108&) |
| IL-1β | Interleukin-1 beta precursor | GO:0007267 : [cell-cell signaling](http://amigo.geneontology.org/cgi-bin/amigo/term-details.cgi?term=GO:0007267&session_id=6194amigo1218010880&)  GO:0006954 : [inflammatory response](http://amigo.geneontology.org/cgi-bin/amigo/term-details.cgi?term=GO:0006954&session_id=6194amigo1218010880&)  GO:0008285 : [negative regulation of cell proliferation](http://amigo.geneontology.org/cgi-bin/amigo/term-details.cgi?term=GO:0008285&session_id=6194amigo1218010880&)  GO:0007165 : [signal transduction](http://amigo.geneontology.org/cgi-bin/amigo/term-details.cgi?term=GO:0007165&session_id=6194amigo1218010880&) |
| IL2RA | Interleukin-2 receptor alpha chain precursor | GO:0008283 : [cell proliferation](http://amigo.geneontology.org/cgi-bin/amigo/term-details.cgi?term=GO:0008283&session_id=3419amigo1218010951&)  GO:0007166 : [cell surface receptor linked signal transduction](http://amigo.geneontology.org/cgi-bin/amigo/term-details.cgi?term=GO:0007166&session_id=3419amigo1218010951&)  GO:0006955 : [immune response](http://amigo.geneontology.org/cgi-bin/amigo/term-details.cgi?term=GO:0006955&session_id=3419amigo1218010951&) |
| IL-3 | Interleukin-3 precursor | GO:0007267 : [cell-cell signaling](http://amigo.geneontology.org/cgi-bin/amigo/term-details.cgi?term=GO:0007267&session_id=6291amigo1218011009&)  GO:0008284 : [positive regulation of cell proliferation](http://amigo.geneontology.org/cgi-bin/amigo/term-details.cgi?term=GO:0008284&session_id=6291amigo1218011009&) |
| IL-8 | Monocyte-derived neutrophil chemotactic factor | GO:0007267 : [cell-cell signaling](http://amigo.geneontology.org/cgi-bin/amigo/term-details.cgi?term=GO:0007267&session_id=8827amigo1216799375&)  GO:0007186 : [G-protein coupled receptor protein signaling pathway](http://amigo.geneontology.org/cgi-bin/amigo/term-details.cgi?term=GO:0007186&session_id=8827amigo1216799375&)  GO:0006954 : [inflammatory response](http://amigo.geneontology.org/cgi-bin/amigo/term-details.cgi?term=GO:0006954&session_id=8827amigo1216799375&)  GO:0007242 : [intracellular signaling cascade](http://amigo.geneontology.org/cgi-bin/amigo/term-details.cgi?term=GO:0007242&session_id=8827amigo1216799375&)  GO:0030155 : [regulation of cell adhesion](http://amigo.geneontology.org/cgi-bin/amigo/term-details.cgi?term=GO:0030155&session_id=8827amigo1216799375&)  GO:0045091 : [regulation of retroviral genome replication](http://amigo.geneontology.org/cgi-bin/amigo/term-details.cgi?term=GO:0045091&session_id=8827amigo1216799375&) |
| TNF-α | Tumor necrosis factor precursor | GO:0006959 : [humoral immune response](http://amigo.geneontology.org/cgi-bin/amigo/term-details.cgi?term=GO:0006959&session_id=1760amigo1216795687&)  GO:0006954 : [inflammatory response](http://amigo.geneontology.org/cgi-bin/amigo/term-details.cgi?term=GO:0006954&session_id=1760amigo1216795687&)  GO:0043123 : [positive regulation of I-kappaB kinase/NF-kappaB cascade](http://amigo.geneontology.org/cgi-bin/amigo/term-details.cgi?term=GO:0043123&session_id=1760amigo1216795687&)  GO:0051092 : [positive regulation of NF-kappaB transcription factor activity](http://amigo.geneontology.org/cgi-bin/amigo/term-details.cgi?term=GO:0051092&session_id=1760amigo1216795687&)  GO:0051023 : [regulation of immunoglobulin secretion](http://amigo.geneontology.org/cgi-bin/amigo/term-details.cgi?term=GO:0051023&session_id=1760amigo1216795687&) |
| TNFSF1 | Lymphotoxin-alpha precursor | GO:0007267 : [cell-cell signaling](http://amigo.geneontology.org/cgi-bin/amigo/term-details.cgi?term=GO:0007267&session_id=359amigo1218010291&) |

**Table 2**: Features of the Host Response: Common Repression

| **Gene Symbol** | **Description** | **GO bio-Function** |
| --- | --- | --- |
| ADAM8 | A disintegrin and metalloproteinase domain 8 | GO:0005887 : [integral to plasma membrane](http://amigo.geneontology.org/cgi-bin/amigo/term-details.cgi?term=GO:0005887&session_id=1158amigo1218011641&) |
| CCR1 | C-C chemokine receptor type 1 | GO:0007155 : [cell adhesion](http://amigo.geneontology.org/cgi-bin/amigo/term-details.cgi?term=GO:0007155&session_id=3150amigo1218011669&)  GO:0007267 : [cell-cell signaling](http://amigo.geneontology.org/cgi-bin/amigo/term-details.cgi?term=GO:0007267&session_id=3150amigo1218011669&)  GO:0007187 : [G-protein signaling, coupled to cyclic nucleotide second messenger](http://amigo.geneontology.org/cgi-bin/amigo/term-details.cgi?term=GO:0007187&session_id=3150amigo1218011669&)  GO:0006955 : [immune response](http://amigo.geneontology.org/cgi-bin/amigo/term-details.cgi?term=GO:0006955&session_id=3150amigo1218011669&)  GO:0006954 : [inflammatory response](http://amigo.geneontology.org/cgi-bin/amigo/term-details.cgi?term=GO:0006954&session_id=3150amigo1218011669&) |
| CD14 | Monocyte differentiation antigen CD14 precursor | GO:0007166 : [cell surface receptor linked signal transduction](http://amigo.geneontology.org/cgi-bin/amigo/term-details.cgi?term=GO:0007166&session_id=9927amigo1218011758&)  GO:0006909 : [phagocytosis](http://amigo.geneontology.org/cgi-bin/amigo/term-details.cgi?term=GO:0006909&session_id=9927amigo1218011758&) |
| CD31 | Platelet endothelial cell adhesion molecule precursor | GO:0030334 : [regulation of cell migration](http://amigo.geneontology.org/cgi-bin/amigo/term-details.cgi?term=GO:0030334&session_id=1647amigo1218012091&)  GO:0042060 : [wound healing](http://amigo.geneontology.org/cgi-bin/amigo/term-details.cgi?term=GO:0042060&session_id=1647amigo1218012091&) |
| CD64 | High affinity immunoglobulin gamma Fc receptor I precursor | GO:0001788 : [antibody-dependent cellular cytotoxicity](http://amigo.geneontology.org/cgi-bin/amigo/term-details.cgi?term=GO:0001788&session_id=5340amigo1218011947&)  GO:0019884 : [antigen processing and presentation of exogenous antigen](http://amigo.geneontology.org/cgi-bin/amigo/term-details.cgi?term=GO:0019884&session_id=5340amigo1218011947&)  GO:0007166 : [cell surface receptor linked signal transduction](http://amigo.geneontology.org/cgi-bin/amigo/term-details.cgi?term=GO:0007166&session_id=5340amigo1218011947&)  GO:0042742 : [defense response to bacterium](http://amigo.geneontology.org/cgi-bin/amigo/term-details.cgi?term=GO:0042742&session_id=5340amigo1218011947&)  GO:0006911 : [phagocytosis, engulfment](http://amigo.geneontology.org/cgi-bin/amigo/term-details.cgi?term=GO:0006911&session_id=5340amigo1218011947&) |
| CYBB | cytochrome b-245, beta polypeptide | GO:0006954 : [inflammatory response](http://amigo.geneontology.org/cgi-bin/amigo/term-details.cgi?term=GO:0006954&session_id=6494amigo1218012157&)  GO:0045087 : [innate immune response](http://amigo.geneontology.org/cgi-bin/amigo/term-details.cgi?term=GO:0045087&session_id=6494amigo1218012157&) |
| FPR | fMet-Leu-Phe receptor | GO:0006928 : [cell motility](http://amigo.geneontology.org/cgi-bin/amigo/term-details.cgi?term=GO:0006928&session_id=5544amigo1218012225&)  GO:0007186 : [G-protein coupled receptor protein signaling pathway](http://amigo.geneontology.org/cgi-bin/amigo/term-details.cgi?term=GO:0007186&session_id=5544amigo1218012225&)  GO:0007188 : [G-protein signaling, coupled to cAMP nucleotide second messenger](http://amigo.geneontology.org/cgi-bin/amigo/term-details.cgi?term=GO:0007188&session_id=5544amigo1218012225&)  GO:0007165 : [signal transduction](http://amigo.geneontology.org/cgi-bin/amigo/term-details.cgi?term=GO:0007165&session_id=5544amigo1218012225&) |
| ITGAX | Leukocyte adhesion receptor p150,95 | GO:0007155 : [cell adhesion](http://amigo.geneontology.org/cgi-bin/amigo/term-details.cgi?term=GO:0007155&session_id=6656amigo1218012329&)  GO:0009887 : [organ morphogenesis](http://amigo.geneontology.org/cgi-bin/amigo/term-details.cgi?term=GO:0009887&session_id=6656amigo1218012329&) |
| NCF1 | Neutrophil cytosol factor 1 | GO:0006968 : [cellular defense response](http://amigo.geneontology.org/cgi-bin/amigo/term-details.cgi?term=GO:0006968&session_id=2363amigo1218012357&)  GO:0005625 : [soluble fraction](http://amigo.geneontology.org/cgi-bin/amigo/term-details.cgi?term=GO:0005625&session_id=2363amigo1218012357&) |
| WASP | Wiskott-Aldrich syndrome protein | GO:0007596 : [blood coagulation](http://amigo.geneontology.org/cgi-bin/amigo/term-details.cgi?term=GO:0007596&session_id=206amigo1218012403&)  GO:0006952 : [defense response](http://amigo.geneontology.org/cgi-bin/amigo/term-details.cgi?term=GO:0006952&session_id=206amigo1218012403&)  GO:0008544 : [epidermis development](http://amigo.geneontology.org/cgi-bin/amigo/term-details.cgi?term=GO:0008544&session_id=206amigo1218012403&)  GO:0006955 : [immune response](http://amigo.geneontology.org/cgi-bin/amigo/term-details.cgi?term=GO:0006955&session_id=206amigo1218012403&) |

**Table 3: The inflammatory genes and their regulators**

| **Gene Name** | **(A)**  **Possible regulators from JASPAR** | **(B) Candidate regulators from Cross-correlation threshold** | **(C) Refined regulators from AIC** |
| --- | --- | --- | --- |
| **ADAM8** | RUNX1,TFAP2A,CREB1,ELK1,GATA2,GATA3,MAX,SP1,SPI1,SPIB,YY1,REL,NFKB1 | CREB1,ELK1,GATA2,GATA3,MAX,SP1,SPI1,SPIB,YY1,NFKB1 | CREB1,ELK1,GATA2,GATA3,MAX,SP1,SPI1,SPIB,YY1,NFKB1 |
| **CCR1** | HLF,IRF1,MEF2A,REL,RELA,RORA,RUNX1,SP1,SPI1,SPIB,YY1 | IRF1,MEF2A,RORA,SP1,SPI1,YY1 | IRF1,MEF2A,RORA,SP1,SPI1,YY1 |
| **CD14** | E2F1,ELK1,GATA2 GATA3,HLF,IRF1,MAX,NFIL3,NFKB1,REL,RUNX1,SP1,SPI1,SPIB,YY1 | E2F1,ELK1,GATA2 GATA3,HLF,IRF1,MAX,NFIL3,NFKB1,RUNX1,SP1,SPI1,YY1 | E2F1,ELK1,GATA2 GATA3,HLF,IRF1,MAX,NFIL3,NFKB1,RUNX1,SP1,SPI1,YY1 |
| **CD31** | E2F1,ELK1,GATA2 GATA3,IRF1,MAX,NFIL3,REL,RUNX1 SP1,SPI1,SPIB,YY1 | E2F1,ELK1,GATA2 GATA3,IRF1,MAX,NFIL3,RUNX1,SP1,SPI1,YY1 | E2F1,ELK1,GATA3 IRF1,MAX,NFIL3,RUNX1,SP1,SPI1,YY1 |
| **CD64** | E2F1,ELK1,GATA2 GATA3,HLF,IRF1,MAX,NFIL3,REL,RELA,RUNX1,SPI1,SPIB,YY1 | E2F1,ELK1,GATA2 GATA3,HLF,IRF1,MAX,NFIL3,RELA,RUNX1,SPI1,SPIB,YY1 | ELK1,GATA2,GATA3,HLF,IRF1,MAX,NFIL3,RELA,RUNX1,SPIB,YY1 |
| **CYBB** | GATA2,GATA3,IRF1,REL,RUNX1,SP1,SPI1,SPIB,SRY,YY1 | GATA2,IRF1,RUNX1,SP1,SPI1,SPIB,YY1 | GATA2,IRF1,SP1,SPI1,SPIB,YY1 |
| **FPR** | CREB1,E2F1,ELK1 GATA2,GATA3,HLF MAX,MEF2A,REL,RORA,RUNX1,SP1,SPI1,SPIB,SRY,YY1 | CREB1,E2F1,ELK1 GATA2,HLF,MAX,MEF2A,RORA,RUNX1,SP1,SPI1,SPIB,YY1 | CREB1,E2F1,ELK1 GATA2,HLF,MAX,MEF2A,RORA,RUNX1,SP1,SPI1,SPIB,YY1 |
| **ITGAX** | CREB1,E2F1,ELK1 GATA2,GATA3,IRF1,NFKB1,RELA,RORA,RUNX1,SP1,SPI1,SPIB,SRY,YY1 | CREB1,E2F1,ELK1 GATA2,GATA3,IRF1,NFKB1,RELA,RORA,RUNX1,SP1,SPI1,YY1 | CREB1,E2F1,ELK1 GATA2,GATA3,IRF1,NFKB1,RELA,RUNX1,SP1,SPI1,YY1 |
| **NCF1** | ELK1,GATA2,GATA3,HLF,MEF2A,SP1 SPI1,SPIB,SRY,YY1 | ELK1,GATA2,GATA3,HLF,MEF2A,SP1 SPI1,SPIB,SRY,YY1 | ELK1,GATA2,GATA3,HLF,MEF2A,SPI1,SPIB,SRY,YY1 |
| **WASP** | CREB1,ELK1,GATA2,GATA3,MAX,NFKB1,REL,RELA,RUNX1,SP1,SPI1,SPIB,SRY,YY1 | CREB1,ELK1,GATA2,GATA3,MAX,NFKB1,RELA,RUNX1 SP1,SPI1,SPIB,YY1 | CREB1,ELK1,GATA3,MAX,RELA,RUNX1,SPI1,SPIB,YY1 |
| **CD40** | E2F1,ELK1,GATA2 GATA3,IRF1,IRF2,MAX,MEF2A,NFIL3,NFKB1,Pbx,REL,RELA,RORA,RUNX1,SP1,SPI1,SPIB,SRY,YY1 | E2F1,ELK1,GATA2 GATA3,IRF1,IRF2,MAX,MEF2A,NFIL3,NFKB1,Pbx,REL,RELA,RORA,RUNX1,SP1,SPI1,SPIB,YY1 | E2F1,ELK1,GATA2 GATA3,IRF1,IRF2,MAX,MEF2A,NFIL3,NFKB1,Pbx,REL,RELA,RORA,RUNX1,SP1,SPI1,SPIB,YY1 |
| **CSF** | E2F1,GATA2,GATA3,IRF1,IRF2,MAX,NFKB1,REL,RELA,RUNX1,SP1,SPI1,SPIB | E2F1,GATA2,GATA3,MAX,NFKB1,RELA,RUNX1,SP1,SPIB | E2F1,GATA2,GATA3,MAX,NFKB1,RELA,RUNX1,SP1,SPIB |
| **IL1A** | E2F1,ELK1,GATA2 GATA3,HLF,MEF2A,NFIL3,Pbx,RUNX1,SPI1,SPIB,SRY,YY1 | E2F1,ELK1,GATA2 GATA3,HLF,MEF2A,NFIL3,Pbx,RUNX1,SPI1,SPIB,YY1 | ELK1,GATA2,GATA3,HLF,MEF2A,NFIL3,Pbx,RUNX1,SPI1,SPIB,YY1 |
| **IL1B** | ELK1,GATA2,GATA3,MEF2A,REL,RELA,SP1,SPI1,SPIB,SRY,YY1 | ELK1,GATA2,GATA3,MEF2A,RELA,SP1,SPI1,YY1 | ELK1,GATA2,GATA3,MEF2A,RELA,SP1,SPI1,YY1 |
| **IL2RA** | CREB1,E2F1,ELK1 GATA2,GATA3,HLF IRF1,MEF2A,NFIL3 REL,RELA,RORA,RUNX1,SPI1,SPIB,SRY,YY1 | CREB1,E2F1,ELK1 GATA2,GATA3,HLF IRF1,MEF2A,NFIL3 RELA,RORA,RUNX1,SPI1,YY1 | CREB1,E2F1,ELK1 GATA2,GATA3,HLF IRF1,MEF2A,NFIL3 RELA,RORA,RUNX1,SPI1,YY1 |
| **IL3** | CREB1,E2F1,ELK1 GATA2,GATA3,HLF IRF1,MEF2A,NFIL3 NFKB1,REL,RELA,RORA,RUNX1,SP1 SPI1,SPIB,SRF,SRY,YY1 | CREB1,E2F1,ELK1 GATA2,GATA3,HLF IRF1,MEF2A,NFIL3 NFKB1,RELA,RORA,RUNX1,SP1,SPI1,SRF,YY1 | CREB1,E2F1,ELK1 GATA2,GATA3,IRF1,MEF2A,NFIL3,NFKB1,RELA,RORA,RUNX1,SP1,SPI1,SRF,YY1 |
| **IL8** | E2F1,ELK1,GATA2 GATA3,HLF,MAX,MEF2A,NFIL3,Pbx,REL,RELA,RORA,RUNX1,SPI1,SPIB,SRY,YY1 | E2F1,ELK1,GATA2 GATA3,HLF,MAX,MEF2A,NFIL3,Pbx,RELA,RORA,RUNX1,SPI1,SPIB,YY1 | E2F1,ELK1,GATA3 HLF,MAX,MEF2A,NFIL3,Pbx,RORA,RUNX1,SPI1,SPIB,YY1 |
| **TNFA** | GATA2,GATA3,MAX,NFKB1,SPI1,SPIB,SRY,YY1 | GATA2,GATA3,MAX,NFKB1,SPI1,SPIB,YY1 | GATA2,GATA3,MAX,NFKB1,SPI1,YY1 |
| **TNFSF1** | CREB1,E2F1,ELK1 GATA2,GATA3,HLF IRF1,MAX,MEF2A REL,RELA,RUNX1 SP1,SPI1,SPIB,YY1 | CREB1,ELK1,GATA2,GATA3,IRF1,MAX,MEF2A,RELA,SP1,SPI1,YY1 | CREB1,ELK1,GATA2,GATA3,IRF1,MAX,MEF2A,RELA,SP1,YY1 |


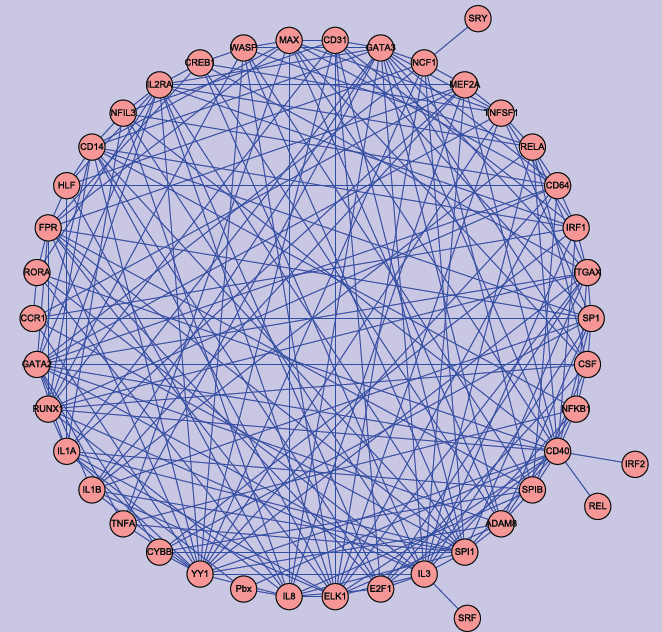
**Figure 1: The gene regulatory network in inflammatory condition.**


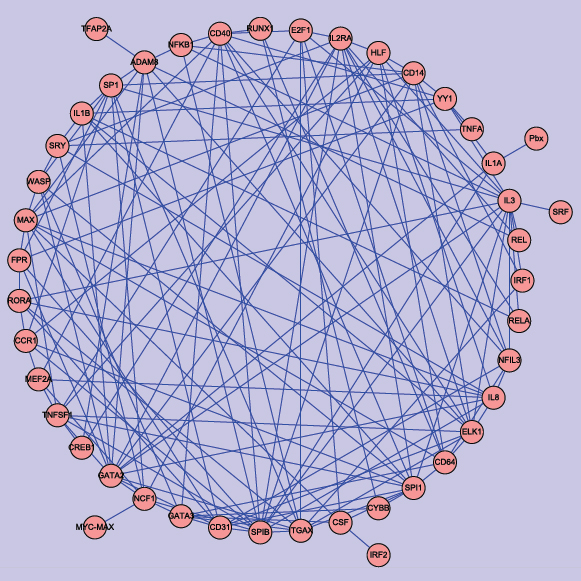
**Figure 2: The gene regulatory network in normal condition.**


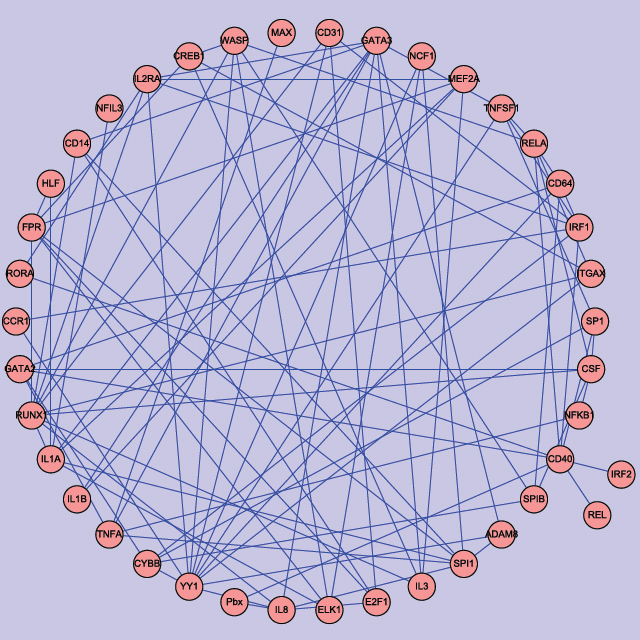
**Figure 3:** **Gene network only in inflammatory condition but not in normal condition.**
